# The benefits of investments to combat HIV, tuberculosis, and malaria for primary healthcare from 2000 to 2023: An economic modeling analysis

**DOI:** 10.1371/journal.pmed.1005036

**Published:** 2026-04-08

**Authors:** Jiaying Stephanie Su, John Stover, Carel Pretorius, Peter Winskill, Sedona Sweeney, Timothy B. Hallett, Nicolas A. Menzies

**Affiliations:** 1 Department of Global Health and Population, Harvard T.H. Chan School of Public Health, Boston, Massachusetts, United States of America; 2 Center for Health Decision Science, Harvard T.H. Chan School of Public Health, Boston, Massachusetts, United States of America; 3 Avenir Health, Glastonbury, Connecticut, United States of America; 4 MRC Centre for Global Infectious Disease Analysis, School of Public Health, Imperial College London, London, United Kingdom; 5 Department of Global Health and Development, Faculty of Public Health Policy, London School of Hygiene and Tropical Medicine, London, United Kingdom; Universitair Medisch Centrum Utrecht, NETHERLANDS, KINGDOM OF THE

## Abstract

**Background:**

Global investments to combat HIV, tuberculosis, and malaria (HTM) have delivered substantial health gains and may have reduced the burden placed by these diseases on the routine health system. We estimated the reduction in primary healthcare (PHC) utilization resulting from the scale-up of HTM services over 2000–2023 in 108 low- and middle-income countries.

**Methods and findings:**

For each disease, we applied established mathematical models to quantify PHC utilization (outpatient visits and inpatient bed-days provided outside of HTM programs) by individuals with symptomatic HIV, tuberculosis, or malaria unable to access HTM-specific services. For each country, we estimated averted PHC utilization by comparing a scenario describing the actual scale-up of HTM services to a counterfactual scenario holding HTM service coverage constant at year 2000 levels. We applied published unit costs to estimate the averted costs resulting from reduced PHC utilization. Over 2000–2023, scale-up of HTM services averted an estimated 6.9 (95% uncertainty interval (UI) [4.4, 10.5]) billion outpatient PHC visits and 3.9 (95% UI [2.5, 5.9]) billion inpatient bed-days, representing US$135 (95% UI [71, 250]) billion in averted costs. These reductions were greatest in sub-Saharan Africa and East Asia and Pacific regions. Across study countries, these reductions represented a median of 4.4% of hospital bed capacity and 1.6% of government health spending in 2023. These percentages were 22.9% and 5.1%, respectively, for low-income countries. Our analysis did not consider changes in PHC services beyond utilization. Also, several inputs were missing in some countries, with missing values estimated using regression imputation.

**Conclusions:**

Over recent decades, sustained investments in HTM services in high-burden settings have averted substantial PHC utilization and associated costs. These benefits should be considered when assessing investment impact.

## Author summary

**Why was this study done?:**

- Vertical disease-control programs addressing HIV, tuberculosis (TB), and malaria have saved millions of lives over the past two decades, but there is little evidence on their impact on routine primary healthcare services.
- Understanding the broader impacts of HIV, TB, and malaria investments is valuable for planning global health financing and service delivery.

**What did the researchers do and find?:**

- Using epidemiological and economic modeling, we estimated the impact of investments to scale-up HIV, TB, and malaria services on primary healthcare utilization and costs for 108 low- and middle-income countries from 2000 to 2023.
- Investments in HIV, TB, and malaria services were estimated to have averted 6.9 billion primary healthcare outpatient visits, 3.9 billion hospital bed-days over 2000–2023, equivalent to US$135 billion in averted costs.
- The largest effects were observed in low-income countries and in the sub-Saharan Africa region.

**What do these findings mean?:**

- Investments in HIV, TB, and malaria disease-control programs not only reduce illness and death but also substantially ease pressure on overstretched health services, freeing up resources for other healthcare.
- These indirect health-system benefits should be considered when governments and donors evaluate the overall value of disease-control programs and plan future funding strategies.
- This study only considered the indirect effects of HIV, TB, and malaria programs on primary healthcare utilization. We did not quantify the direct health benefits of these programs, or assess the consequence for primary healthcare services beyond utilization. Some country-level inputs were estimated with regression imputation. Results for individual countries should be interpreted with caution.

## Abstract 3

In an economic modeling analysis study, Jiaying Stephanie Su and colleagues investigate the reduction in primary health care utilization resulting from investments to combat HIV, tuberculosis and malaria HTM over 2000–2023 in 108 low- and middle-income countries.

## Introduction

Since 2000, global efforts to combat human immunodeficiency virus (HIV), tuberculosis (TB), and malaria have reduced the incidence of HIV by 54%, TB by 26%, and malaria by 24% [1–3]. These achievements would not have been possible without sustained investments to scale up services to prevent and treat these diseases. These investments have been made by domestic governments and international partners, with funding provided through bilateral mechanisms such as the U.S. President's Emergency Plan for AIDS Relief and multilateral mechanisms such as the Global Fund to Fight AIDS, Tuberculosis, and Malaria. In 2023, US$30 billion was spent by both domestic and international partners to advance HIV, TB, and malaria elimination in low- and middle-income countries (LMICs) [1–3], reflecting global commitment to ending these diseases as public health threats.

Analyses of investments to address HIV, TB, and malaria (HTM) have typically compared the health benefits of these interventions to the resources required to scale and maintain HTM-specific services [4–6]. However, less attention has been directed toward the implications for primary healthcare (PHC), except when these services are involved in providing HTM services. Although normative guidelines for economic evaluation stress the value of considering all consequences [7], evidence regarding the impact of HTM investments on health systems remains inconclusive. Concerns have been raised that global health initiatives may distort incentives within the health system and undermine services for non-targeted conditions, while reviews of empirical studies have documented both positive and negative effects [8,9]. These studies highlight the need to quantify the implications of disease-specific investments on health systems, comparing possible outcomes under different levels of disease-specific investment. One way in which HTM investments could impact health systems is through changes in demand for PHC services. These changes in demand could arise through multiple mechanisms. First, disease-specific care provided through HTM programs could reduce the number of individuals experiencing untreated symptomatic disease, potentially crowding out care that would otherwise be sought from PHC providers. Second, improved HTM care prevents progression to advanced illness, reducing the need for inpatient care. Third, better HTM prevention and treatment can interrupt disease transmission, preventing new infections so that fewer symptomatic individuals require care at all. Each of these mechanisms could reduce PHC demand and free up healthcare resources for other uses, which would be particularly valuable in countries with limited healthcare resources.

In the context of evolving global priorities and growing fiscal constraints, it is important to understand not only the direct outcomes of disease-specific investments but also their broader implications for health system capacity and expenditure. In this study, we estimated the impact of investments to scale-up HIV, TB, and malaria services over 2000–2023 on PHC utilization and costs outside of HTM-specific programs. We conducted this analysis for 108 LMICs with a high burden of HIV, TB, or malaria.

## Methods

### Analytic summary

We considered individuals with or at risk of HIV, TB, or malaria, residing in LMICs defined as having a high burden of one or more of these conditions by WHO or UNAIDS [1–3], and for which epidemiological outcomes were available from validated disease simulation models used in an earlier modeling exercise [10]. We identified 108 countries for HIV, 51 countries for TB, and 55 countries for malaria in our analyses, representing 94% of global HIV prevalence, 92% of global TB incidence, and 90% of global malaria incidence in 2023. Countries included in this study are listed in Table A in S1 Appendix.

For each disease, we compared two scenarios covering years 2000–2023: an ***actual scenario*** reflecting the observed scale-up of HTM services in each country since year 2000, and a counterfactual ***constant coverage scenario*** holding HTM service coverage fixed at year 2000 levels. In the counterfactual scenario, reductions in HTM service coverage were assumed to reflect lower funding from all sources, and these funding sources were not considered separately. In all scenarios, general population rates of outpatient and inpatient PHC utilization were allowed to vary between countries and grow over time based on published estimates [11]. The primary outcomes estimated in this study were the numbers of PHC outpatient visits, PHC inpatient bed-days, and PHC costs averted through the scale-up of HTM services in modeled countries over the study period. For each scenario, PHC utilization and costs were estimated from modeled estimates of the number of individuals with symptomatic HIV, tuberculosis, or malaria not receiving effective disease-specific care through HTM programs. Estimates of averted PHC utilization and costs were calculated from the difference in each outcome between actual and counterfactual scenarios, and are reported by disease, country, year, World Bank region, and income classification. To contextualize these findings, we also expressed averted inpatient utilization as a percentage of national hospital bed capacity, and averted PHC costs as a percentage of domestic government health expenditures [12,13]. In sensitivity analyses, we assessed an additional counterfactual (***no coverage scenario***) in which HTM service coverage was reduced to zero from 2000 onward throughout the study period. All scenarios were assumed to be identical prior to 2000.

This study is reported as per the Consolidated Health Economic Evaluation Reporting Standards (CHEERS) guideline [14].

### Estimation of disease epidemiology

We obtained epidemiological outcomes for each disease and scenario from modeled analyses undertaken as part of the investment case for the Global Fund's 8th replenishment [15]. These models included the *Goals Model* for HIV, the *Global Portfolio TB Model* for TB, and the *malariasimulation* model for malaria, for which full methodological details have been described in published sources [16–18]. For the ***actual scenario***, models incorporated the historical scale-up of HTM services (defined as the fraction of eligible individuals receiving each service) as inputs, and were calibrated to epidemiological estimates published by WHO and UNAIDS [1–3]. Counterfactual scenarios represented hypothetical changes in HTM service coverage over the study period, with all other modeled mechanisms (e.g., demographic processes, intervention quality) following the assumptions of the ***actual scenario***. For TB, we used the modeled results from the *Global Portfolio TB Model* (29 countries) to train a regression-based emulator and simulate epidemiological outcomes for an additional 22 high TB burden countries (technical details in S1 Appendix) [2]. This increased the fraction of global TB incidence represented in our study from 81% to 92%.

### Estimation of averted PHC utilization

Using the epidemiological estimates produced by the disease simulation models, we calculated the number of individuals with symptomatic HIV, TB, or malaria that were unable to access effective HTM-specific care under each scenario. For HIV, this was operationalized as the annual number of person-years lived by persons with HIV not receiving antiretroviral therapy (ART). For TB and malaria, this was operationalized as the annual number of individuals developing each disease that did not receive disease-specific care. For each disease, we computed the differences in these quantities between the actual and counterfactual scenarios over the study period. To calculate averted PHC utilization, we applied estimates of the additional outpatient care (number of clinic visits) and inpatient care (number of hospital bed-days) received by affected individuals, as compared to individuals not experiencing these conditions. We purposely did not include changes in utilization and associated costs within HTM programs, as these outcomes have been reported by other analyses [19]. We conducted analyses separately for each country and disease. Methods specific to each disease are described below. Values and sources for analytic inputs are provided in S1 Appendix.

#### HIV.

For each country and scenario, the number of person-years lived with untreated HIV was stratified by age group (0–4 years, 5–14 years, and ≥15 years) and CD4 cell count category (<50, 50–99, 100–199, 200–249, 250–349, 350–499, and ≥500 cells/mm^3^), producing 21 strata. For each stratum, we calculated the difference between actual and counterfactual scenarios, and multiplied this difference by an estimate of the excess PHC utilization per person-year (defined as the additional utilization for an individual in each stratum compared to individuals without HIV). Estimates of averted PHC outpatient visits (Equation 1) and inpatient bed-days (Equation 2) were calculated for each country and year by summing across age groups and CD4 cell count categories.


$${Avertedoutpatientvisits}_{c,y}=\sum_{a,d}\left[\Delta \overset{HIV}{\underset{c,y,a,d}{PY}}\times \overset{op}{\underset{c,y,a}{U}}\times (\overset{op\_HIV}{\underset{d}{RR}}-\text{1})\right]$$ (1)

$${Avertedinpatientbeddays}_{c,y}=\sum_{a,d}\left[\Delta \overset{HIV}{\underset{c,y,a,d}{PY}}\times \overset{ip}{\underset{c,y,a}{U}}\times (\overset{ip\_HIV}{\underset{d}{RR}}-\text{1})\times LOS\right]$$ (2)

where $\Delta \overset{HIV}{\underset{c,y,a,d}{PY}}$ is defined as the difference in the number of person-years lived with untreated HIV between actual and counterfactual scenarios for country *c*, year *y*, age group *a*, and CD4 category *d*; $\overset{op}{\underset{c,y,a}{U}}$ is the expected number of outpatient visits per person-year for individuals in the general population by country, year, and age group; $\overset{op\_HIV}{\underset{d}{RR}}$ is the rate ratio of outpatient visits for individuals with untreated HIV in CD4 stratum *d* relative to the general population; $\overset{ip}{\underset{c,y,a}{U}}$ is the number of hospitalization episodes per person-year for individuals in the general population; $\overset{ip\_HIV}{\underset{d}{RR}}$ is the rate ratio of hospitalization episodes by individuals with untreated HIV in CD4 stratum *d* relative to the general population; and LOS is the average length of hospitalization per episode, in days. A flowchart of the model structure is provided in Fig A1 in S1 Appendix. Inputs for Equations 1 and 2 were derived from the HIV epidemiological model ($\Delta \overset{HIV}{\underset{c,y,a,d}{PY}}$), from published sources ($\overset{op}{\underset{c,y,a}{U}}$, $\overset{ip}{\underset{c,y,a}{U}}$, and LOS), or from regression models fit to published estimates ($\overset{op\_HIV}{\underset{d}{RR}}$ and $\overset{ip\_HIV}{\underset{d}{RR}}$, details reported in S1 Appendix). Values and sources for each input parameter are reported in Table B1 in S1 Appendix.

#### TB.

For each country and scenario, we stratified the annual number of individuals developing TB according to whether or not these individuals received effective TB treatment (“treated” and “untreated” cases respectively), and calculated the difference in these values between actual and counterfactual scenarios. For treated cases we assumed there would be a period of additional outpatient PHC utilization (as compared to individuals without TB) between the development of symptomatic TB and the initiation of treatment. We multiplied the difference in treated cases between scenarios by an estimate of the additional outpatient visits per treated case. For untreated cases, we assumed there would be additional outpatient PHC utilization between the development of symptomatic TB and the end of the TB episode (death or self-cure), and multiplied the difference in untreated cases between scenarios by an estimate of the additional outpatient visits per untreated case. We summed results for treated and untreated TB to estimate total averted outpatient utilization (Equation 3). We also assumed there would be excess inpatient PHC utilization for individuals dying with untreated TB. To estimate averted inpatient utilization, we multiplied the difference in untreated TB cases between scenarios by an estimate of case fatality for these individuals and the average duration of hospitalization before death (Equation 4).


$$\begin{array}{cc} & {Avertedoutpatientvisits}_{c,y}=\sum \left[\Delta \overset{tx\_TB}{\underset{c,y}{C}}\times \overset{op}{\underset{c,y,a}{U}}\times (\overset{op\_TB}{\underset{d}{RR}}-\text{1})\times D^{predx}\right]\\ & +\sum \left[\Delta \overset{notx\_TB}{\underset{c,y}{C}}\times \overset{op}{\underset{c,y,a}{U}}\times (\overset{op\_TB}{\underset{d}{RR}}-\text{1})\times D^{notx}\right)] \end{array}$$ (3)

$${Avertedinpatientbeddays}_{c,y}=\sum \left(\Delta \overset{notx\_TB}{\underset{c,y}{C}}\times CFR\times {LOS}^{TB}\right)$$ (4)

where $\Delta \overset{tx\_TB}{\underset{c,y}{C}}$ is defined as the difference in the number of treated TB cases between actual and counterfactual scenarios for country *c* and year *y*; $\overset{op}{\underset{c,y,a}{U}}$ is the expected number of outpatient visits per person-year for individuals in the general population by country, year, and age group; $\overset{op\_TB}{\underset{d}{RR}}$ is the rate ratio of outpatient visits for individuals with untreated TB relative to the general population; $D^{predx}$ is the duration of symptomatic TB prior to treatment for treated TB cases;$\Delta \overset{notx\_TB}{\underset{c,y}{C}}$ is the difference in the number of untreated TB cases between actual and counterfactual scenarios for country *c* and year *y*; $D^{notx}$ is the duration of symptomatic TB for untreated cases; CFR is the case fatality rate for untreated TB; and ${LOS}^{TB}$ is the average length of hospital stay for individuals dying with untreated TB. A flowchart of the model structure is provided in Fig A2 in S1 Appendix. Inputs for Equations 3 and 4 were derived from the TB epidemiological model ($\Delta \overset{tx\_TB}{\underset{c,y}{C}}$ and$\Delta \overset{notx\_TB}{\underset{c,y}{C}}$) and from published sources ($\overset{op}{\underset{c,y,a}{U}}$, $\overset{op\_TB}{\underset{d}{RR}}$, $D^{predx}$, and $D^{notx}$). Values and sources for each input parameter are reported in Table B2 in S1 Appendix.

#### Malaria.

For each country and scenario, we calculated the difference between actual and counterfactual scenarios in the annual number of individuals developing symptomatic malaria without receiving effective malaria-specific treatment. To calculate averted outpatient visits, we multiplied this difference by the proportion of individuals with symptomatic malaria who seek care and by the number of outpatient visits per case (Equation 5). To calculate averted inpatient bed-days, we calculated the difference in malaria cases between scenarios, stratified into uncomplicated and severe cases, and multiplied these by the expected proportion hospitalized for each type of case. We summed these values and subtracted the estimated number of hospitalizations for individuals with malaria receiving disease-specific treatment, and then multiplied the resulting estimate by the average duration of hospitalization (Equation 6).


$${Avertedoutpatientvisits}_{c,y}=\sum \left(\Delta \overset{notx\_M}{\underset{c,y}{C}}\times \overset{care}{\underset{c,y}{P}}\times U^{case}\right)$$ (5)

$${Avertedinpatientbeddays}_{c,y}=\sum [(\Delta \overset{unc\_M}{\underset{c,y}{C}}\times \overset{unc}{\underset{c}{P}}+\Delta \overset{sev\_M}{\underset{c,y}{C}}\times \overset{sev}{\underset{c}{P}}-\Delta \overset{hosp\_M}{\underset{c,y}{C}})\times U^{hosp}]$$ (6)

where$\Delta \overset{notx\_M}{\underset{c,y}{C}}$ is defined as the difference in the number of untreated malaria episodes between actual and counterfactual scenarios for country *c* and year *y*; $\overset{care}{\underset{c,y}{P}}$ is the proportion of individuals with symptomatic malaria that seek care; $U^{case}$ is the number of outpatient visits per malaria case; $\Delta \overset{unc\_M}{\underset{c,y}{C}}$ is the difference in the number of uncomplicated malaria cases between actual and counterfactual scenarios for country *c* and year *y*;$\overset{unc}{\underset{c}{P}}$ is the proportion of uncomplicated malaria cases that are hospitalized; $\Delta \overset{sev\_M}{\underset{c,y}{C}}$ is the difference in the number of severe malaria cases between actual and counterfactual scenarios for country *c* and year *y*; $\overset{sev}{\underset{c}{P}}$ is the number of hospitalizations per severe malaria case;$\Delta \overset{hosp\_M}{\underset{c,y}{C}}$ is the difference between actual and counterfactual scenarios in the number of malaria cases hospitalized following appropriate treatment for country *c* and year *y*; and $U^{hosp}$ is the number of inpatient bed-days per hospitalized malaria case. A flowchart of the model structure is provided in Fig A3 in S1 Appendix. Inputs for Equations 5 and 6 were derived from the malaria epidemiological model ($\Delta \overset{notx\_M}{\underset{c,y}{C}}$, $\Delta \overset{unc\_M}{\underset{c,y}{C}}$, $\Delta \overset{sev\_M}{\underset{c,y}{C}}$, and $\Delta \overset{hosp\_M}{\underset{c,y}{C}}$), from published sources ($U^{case}$ and $U^{hosp}$), or from regression models fit to published estimates ($\overset{care}{\underset{c,y}{P}}$, $\overset{unc}{\underset{c}{P}}$, and $\overset{sev}{\underset{c}{P}}$, details reported in S1 Appendix). Values and sources for each input parameter are reported in Table B3 in S1 Appendix.

### Estimation of averted PHC costs

We estimated the averted costs associated with reduced PHC utilization by applying country-specific unit costs to the estimates of averted outpatient visits and inpatient bed-days. Unit costs were retrieved from WHO-CHOICE estimates and adjusted for inflation to reflect nominal U.S. dollars by year [20–22].

### Sensitivity analyses

We used probabilistic sensitivity analysis to quantify uncertainty in analytic outcomes [23]. We specified probability distributions for each uncertain parameter (Table B in S1 Appendix), and drew 1,000 parameter samples using a Latin hypercube design. We used these samples to generate 1,000 estimates for each study outcome and calculated equal-tailed 95% uncertainty intervals (UIs) from the distribution of results. We calculated partial rank correlation coefficients (PRCCs) to assess the relative importance of individual parameters in determining total averted costs [24].

We also evaluated how results changed under three sets of alternative analytic assumptions. In the first of these alternative specifications, we adopted a non-linear cost function that allowed for economies of scale, in which the unit cost per outpatient visit and per inpatient bed-day was assumed to decrease as utilization increases. This was operationalized as an elasticity relationship, in which the average unit cost was assumed to be proportional to exp(ln(utilization)*b). In this relationship, a negative value of *b* allows for reductions in the unit cost for higher levels of utilization. For parameter *b*, we used a value of −0.158 (−0.276, −0.041) for outpatient utilization and −0.148 (−0.276, −0.020) for inpatient utilization, based on published econometric analyses [21,25].

In the second alternative specification, we modified the cost function from the main analysis to explore the additional costs of relaxing capacity constraints with higher levels of PHC utilization (i.e., diseconomies of scale), as might be required for infrastructure investments and staff training. To do so, we extracted data on the distribution of current facility capacity utilization collected as part of a multi-country cost study [26]. We assumed that any increase in utilization would affect all facilities proportionally, and recalculated capacity utilization for the higher utilization level estimated for the counterfactual scenarios. For facilities in which these revised capacity utilization estimates exceeded 100%, we assumed the excess utilization would require additional fixed costs to expand capacity. This was calculated as a 62% markup on the original unit costs, applied to utilization above 100% of the original capacity [26].

In the third alternative specification, we modified the calculation of averted utilization to consider the consequences of reduced HTM mortality, which could lead to ongoing PHC utilization (for reasons other than HTM) by individuals who would have otherwise died of HIV, TB, or malaria. To do so, we adjusted the equations that calculated averted utilization in the main analysis (Equations 1–6) by subtracting the difference in utilization among individuals living without HIV, TB, or malaria between the actual and counterfactual scenarios. This difference in utilization was estimated by calculating the difference in the number of person-years lived by HTM-negative individuals ($\Delta \overset{no\_HTM}{\underset{c,y}{PY}}$) between scenarios, and multiplying this by the annual PHC utilization rate for the general population ($\overset{op}{\underset{c,y,a}{U}}$ for outpatient visits and $\overset{ip}{\underset{c,y,a}{U}}$ ×LOS for inpatient bed-days). For HIV, we calculated $\Delta \overset{no\_HTM}{\underset{c,y}{PY}}$ from the annual numbers of HIV–negative individuals produced by the HIV epidemiological model. For TB and malaria, we first computed the difference in disease-specific deaths between scenarios by country and year from the respective epidemiological models, and distributed these deaths across age groups using published estimates [27]. We then convolved the age-specific time series of incremental deaths with survival estimates derived from general population life tables for each country (Equation 7) [28].


$$\Delta PYc,yno\_HTM=\sum_{a}\sum t=y_{\text{0}}y\left[\Delta D_{c,t}\times qa,cage\times y-tp_{a,c}\right]$$ (7)

where $\Delta \overset{no\_HTM}{\underset{c,y}{PY}}$ is defined as the difference in the number of person-years lived between actual and counterfactual scenarios due to averted HTM deaths in country *c* and year *y*; $\Delta D_{c,t}$ is the difference in disease-specific deaths between actual and counterfactual scenarios for country *c* and year *t*; $\overset{age}{\underset{a,c}{q}}$ is the proportion of disease-specific deaths in age group *a* and country *c*, and _*y*−*t*_*p*_*a,c*_ is the proportion of individuals with s*t*arting age *a* that will still be alive after *y*−*t* years, based on background mortality rates for country *c*. Finally, we applied country-specific unit costs to estimate the averted costs associated with the averted PHC utilization under this alternative specification.

The results of these three alternative analytic specifications were compared to the results of the main analysis to understand their impact on the study findings.

## Results

### Freed-up primary healthcare resources

Over 2000–2023, we estimated substantial reductions in PHC utilization due to the scale-up of HIV, TB, and malaria services across the 108 LMICs included in the analysis. In total, 6.9 (95% UI [4.4, 10.5]) billion outpatient PHC visits and 3.9 (95% UI [2.5, 5.9]) billion inpatient bed-days were averted (Table 1). This reduction in utilization was equivalent to US$135 (95% UI [71, 250]) billion in averted costs. In the sub-Saharan Africa (SSA) region, where reductions in utilization were greatest for HIV and malaria, the scale-up of HIV services was estimated to have averted 0.9 (95% UI [0.6, 1.3]) billion outpatient visits and 1.1 (95% UI [0.4, 2.2]) billion inpatient bed-days over the study period, translating into US$43 (95% UI [13, 113]) billion in averted costs (Table C in S1 Appendix). For malaria, the scale-up of services in SSA was estimated to have averted 2.3 (95% UI [1.3, 3.6]) billion outpatient visits and 0.3 (95% UI [0.2, 0.6]) billion inpatient bed-days, corresponding to US$4.9 (95% UI [2.8, 7.8]) billion in averted costs. Scale-up of TB services contributed a notable portion of utilization reductions in the East Asia and Pacific region, averting 1.7 (95% UI [0.6, 3.4]) billion outpatient visits and 1.0 (95% UI [0.6, 1.7]) billion inpatient bed-days, representing US$53 (95% UI [19, 113]) billion in averted costs between 2000 and 2023.

**Table 1:** Averted primary healthcare utilization and costs due to scale-up HIV, TB, and malaria services, for actual scenario compared to constant coverage scenario, 2000–2023.

	Averted utilization, millions		Averted costs (US$), millions		
	Outpatient visits	Inpatient bed-days	Outpatient	Inpatient	Total
Total	6,911 (4,353, 10,532)	3,892 (2,495, 5,914)	20,507 (9,062, 46,780)	114,279 (53,403, 228,637)	134,787 (71,025, 250,281)
Disease					
HIV	1,188 (757, 1,734)	1,352 (516, 2,710)	4,049 (1,930, 7,688)	50,130 (14,685, 124,045)	54,179 (17,981, 129,624)
TB	3,289 (1,090, 6,658)	2,146 (1,177, 3,526)	13,394 (3,296, 38,136)	61,336 (24,752, 125,018)	74,730 (34,373, 143,816)
Malaria	2,435 (1,369, 3,803)	395 (184, 767)	3,065 (1,455, 5,424)	2,813 (1,188, 6,048)	5,878 (3,357, 9,531)
World Bank region					
East Asia and Pacific	1,826 (704, 3,611)	1,126 (651, 1,825)	10,350 (1,762, 35,079)	48,904 (16,245, 108,235)	59,254 (22,358, 122,284)
Europe and Central Asia	206 (85, 394)	95 (57, 144)	905 (304, 2,180)	3,411 (1,703, 6,089)	4,317 (2,361, 7,169)
Latin America and Caribbean	245 (131, 426)	156 (91, 249)	823 (308, 1,798)	4,357 (1,829, 8,643)	580 (2,472, 9,584)
Middle East and North Africa	5.2 (3.5, 7.3)	3.8 (1.6, 7.3)	28 (12, 55)	170 (59, 385)	197 (80, 421)
South Asia	789 (365, 1,461)	594 (357, 936)	1,241 (262, 3,384)	5,887 (2,076, 13,094)	7,128 (2,846, 14,646)
Sub-Saharan Africa	3,840 (2,582, 5,354)	1,918 (1,146, 3,141)	7,161 (3,749, 12,665)	51,549 (18,005, 125,456)	58,710 (25,214, 135,051)
Income classification					
Low income	1,914 (1,234, 2,766)	624 (412, 920)	1,648 (847, 2,725)	2,436 (1,343, 4,392)	4,085 (2,661, 6,206)
Lower middle income	2,697 (1,693, 4,018)	1,431 (929, 2,147)	4,784 (2,425, 8,619)	15,087 (8,365, 26,003)	19,871 (12,599, 31,425)
Upper middle income	2,301 (1,027, 4,231)	1,837 (1,059, 2,902)	14,075 (3,896, 39,006)	96,756 (39,850, 203,777)	110,831 (51,968, 221,045)
Time period					
2000–2009	864 (535, 13,014)	504 (327, 755)	1,595 (811, 3,167)	10,200 (5,130, 18,301)	11,795 (6,522, 20,529)
2010–2019	3,703 (2,353, 5,573)	2,066 (1,323, 3,216)	10,743 (4,917, 23,857)	61,704 (28,707, 124,331)	72,447 (38,436, 134,776)
2020–2023	2,344 (1,450, 3,547)	1,323 (824, 2,044)	8,169 (3,296, 19,746)	42,376 (19,458, 84,111)	50,545 (26,424, 94,321)

Values in parentheses represent 95% uncertainty intervals. Cost values represent nominal U.S. dollars. Actual scenario represents the observed scale-up of HIV, TB, and malaria services over the study period. Constant coverage scenario represents a counterfactual with coverage of HIV, TB, and malaria services held constant at the year 2000 levels for each country over the study period. HIV, human immunodeficiency virus; TB, tuberculosis.

**Fig 1:**
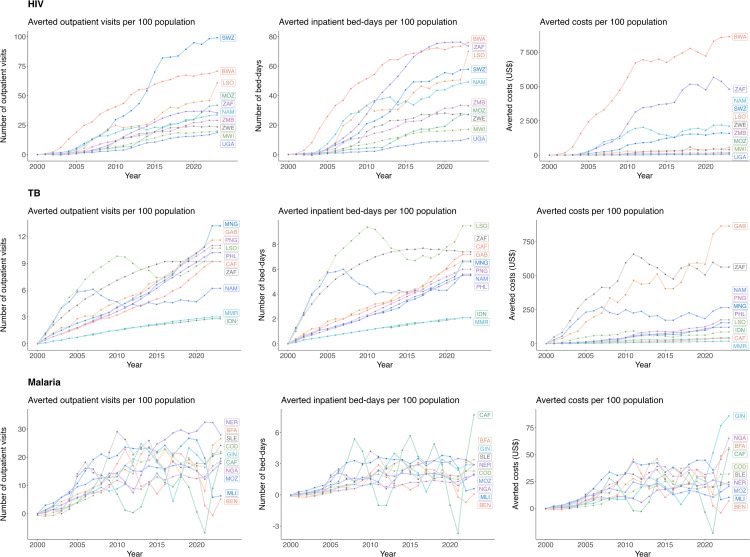
Averted primary healthcare utilization and associated averted costs due to scale-up of HIV, TB, and malaria services from 2000 to 2023, by year, country, and disease. Results shown for 10 modeled countries with the highest HIV prevalence, TB incidence rate, and malaria incidence rate in 2023 [40–42]. Cost values represent nominal U.S. dollars. HIV, human immunodeficiency virus; TB, tuberculosis. Three-letter acronyms in figure (e.g., IDN, ZAF) represent country ISO3 codes.

The magnitude of averted utilization and costs increased over the study period, with 2020–2023 alone accounting for 34% of total averted utilization and 37% of total averted costs. Fig 1 presents time trends for these outcomes for countries with the highest burdens of each disease. In 2023, per 100 population, nearly 100 HIV-related outpatient visits were averted in Eswatini, and close to 80 bed-days were averted in Botswana and South Africa. Total averted costs per 100 population reached over US$7,500 in Botswana. Similar trends were observed for TB and malaria, with the greatest reductions in PHC utilization and associated averted costs estimated for the final years of the time-series. Although the estimates of averted utilization and costs continued to rise over time, we saw a deceleration in the growth of these outcomes for most countries toward the end of the study period.

### Impact on healthcare capacity

**Fig 2:**
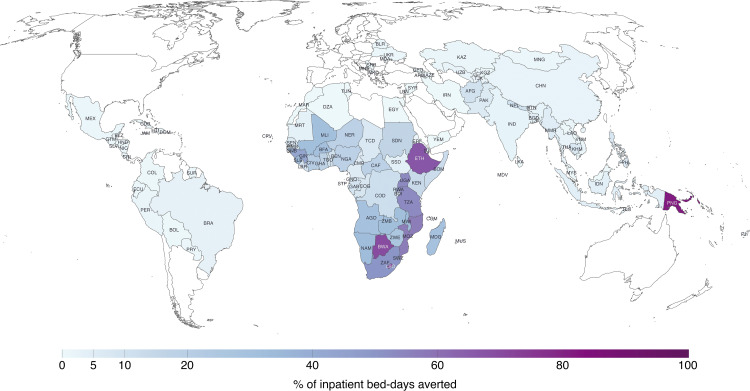
Averted inpatient bed-days due to scale-up of HIV, TB, and malaria services, as a percentage of national hospital bed capacity, 2000–2023. Countries where averted inpatient utilization was over 50% of national hospital bed capacity: Papua New Guinea, Botswana, Lesotho, Ethiopia, Mozambique, Tanzania, Uganda, and South Africa. HIV: human immunodeficiency virus; TB: tuberculosis. Three-letter acronyms in figure (e.g., IDN, ZAF) represent country ISO3 codes. The world map was obtained from Natural Earth, accessed via the *rnaturalearth* R package (*ne_countries, scale = “medium”*). (https://www.naturalearthdata.com). Natural Earth datasets are provided in the public domain (license: https://www.naturalearthdata.com/about/terms-of-use/).

We compared the estimates of averted inpatient bed-days to published estimates of hospital bed capacity for each country, to assess the magnitude of averted utilization relative to existing health system capacity (Fig 2). For 22 countries, the reduction in bed-days across all three diseases represented over 20% of hospital bed capacity over the study period. This impact was especially pronounced in the SSA region, where estimated reductions in hospital utilization were over 50% of national capacity for eight countries (Papua New Guinea, Botswana, Lesotho, Ethiopia, Mozambique, Tanzania, Uganda, and South Africa). Across all SSA countries, the median reduction was 18.0% (interquartile range (IQR) [7.8, 34.5]) over the entire study period and 31.8% (IQR [9.2, 54.8]) for 2023 (Table 2). Averted inpatient utilization represented 22.9% (IQR [8.7, 53.2]) of hospital capacity in low-income countries for 2023, which was almost 4 times greater than the 6.3% (IQR [1.2, 22.3]) in lower-middle-income countries and 13 times the percentages (1.7%, IQR [0.5, 3.5]) in upper-middle-income countries.

**Table 2:** Estimates of averted inpatient utilization as a percentage of national hospital bed capacity and averted costs as a percentage of domestic government health spending, for actual scenario compared to constant coverage scenario, 2000–2023.

	Number of countries included in analysis	Averted inpatient bed-days (% of national hospital bed capacity)		Averted costs (% of domestic government health spending)	
		Whole period (2000–2023)	Final year (2023)	Whole period (2000–2023)	Final year (2023)
Total	108	2.7 (0.6, 14.1)	4.4 (1.1, 21.6)	1.3 (0.1, 4.7)	1.6 (0.2, 5.6)
Disease					
HIV	108	0.7 (0.2, 3.0)	1.3 (0.4, 5.6)	0.2 (0.05, 0.8)	0.3 (0.07, 1.5)
TB	51	4.9 (1.8, 9.6)	7.2 (3.0, 16.1)	1.5 (0.7, 2.1)	2.4 (0.9, 3.7)
Malaria	55	3.2 (0.2, 6.9)	3.8 (0.1, 8.0)	1.3 (0.1, 3.2)	1.5 (0.07, 3.8)
World Bank region					
East Asia and Pacific	13	2.3 (0.4, 5.1)	3.0 (0.7, 7.6)	0.7 (0.3, 1.3)	0.7 (0.3, 1.2)
Europe and Central Asia	13	0.7 (0.2, 1.5)	2.3 (0.7, 4.0)	0.8 (0.2, 2.1)	1.3 (0.3, 3.6)
Latin America and Caribbean	18	0.9 (0.5, 1.1)	1.5 (0.9, 2.3)	0.1 (0.09, 0.2)	0.2 (0.08, 0.3)
Middle East and North Africa	9	0.1 (0.04, 0.14)	0.2 (0.1, 0.5)	0.03 (0.02, 0.05)	0.08 (0.05, 0.1)
South Asia	8	3.6 (0.1, 7.1)	4.9 (0.3, 11.5)	0.9 (0.04, 1.5)	1.0 (0.06, 2.3)
Sub-Saharan Africa	47	18.0 (7.8, 34.5)	31.8 (9.2, 54.8)	6.1 (2.2, 8.8)	9.5 (2.7, 19.8)
Income classification					
Low income	25	16.3 (8.9, 33.7)	22.9 (8.7, 53.2)	4.3 (1.8, 8.8)	5.1 (2.7, 14.1)
Lower middle income	44	4.5 (0.8, 14.4)	6.3 (1.2, 22.3)	1.4 (0.3, 4.2)	2.0 (0.4, 6.3)
Upper middle income	39	0.9 (0.3, 1.8)	1.7 (0.5, 3.5)	0.2 (0.09, 0.8)	0.3 (0.08, 1.2)

Point estimates represent median across modeled countries. Values in parentheses represent interquartile range (25th and 75th percentiles) of country-level values. HIV, human immunodeficiency virus; TB, tuberculosis.

### Impact on healthcare spending

**Fig 3:**
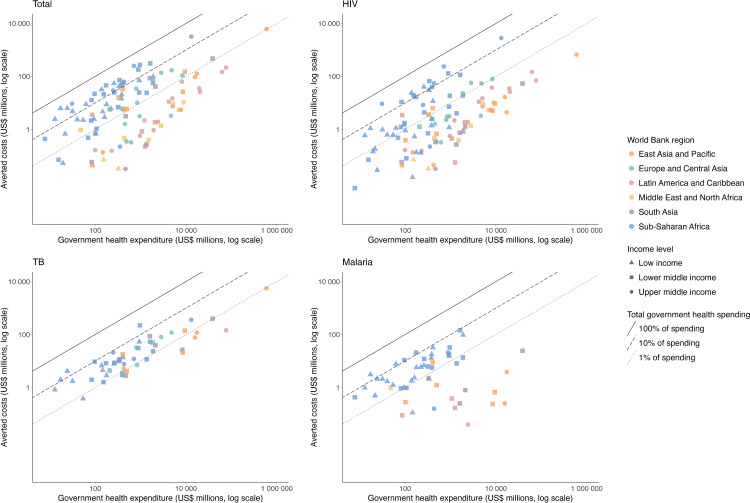
Averted costs due to scale-up of HIV, TB, and malaria services as a percentage of domestic government health spending by country in 2023. Plotted values represent nominal U.S. dollars. HIV, human immunodeficiency virus; TB, tuberculosis.

To put the scale of financial benefits into perspective, we expressed the averted PHC costs as a fraction of published estimates of domestic government health expenditures. Across modeled countries, estimated averted costs represented a median of 1.3% (IQR [0.1, 4.7]) of total government health spending over the whole study period and 1.6% (IQR [0.2, 5.6]) for 2023 (Table 2). These percentages were higher for low-income countries (median in 2023: 5.1%, IQR [2.7, 14.1]) compared to lower-middle-income countries (median in 2023: 2.0%, IQR [0.4, 6.3]) and upper-middle-income countries (median in 2023: 0.3%, IQR [0.08, 1.2]). Fig 3 reports averted costs as a percentage of domestic government health spending across countries to contextualize the magnitude of our results. We observed that averted costs accounted for 10% of domestic government health spending for most countries in the SSA region, as reflected by values clustering around the 10% reference line. Specifically, 12 out of 47 countries in this region exhibited averted costs surpassing 20% of their government health spending in 2023.

### Sensitivity analyses

Tables D and E in S1 Appendix report results for the ***actual scenario*** compared to the ***no coverage scenario***, which assumed no HTM services after year 2000. As expected, these results were larger in magnitude than those from the main analysis. An estimated 18.2 (95% UI [10.4, 29.8]) billion outpatient visits and 10.1 (95% UI [6.3, 15.6]) billion inpatient bed-days were averted, 2.6 times greater than the values reported in the main analysis. Averted costs totaled US$357 (95% UI [184, 651]) billion over the study period, 2.6 times higher than estimated in the main analysis.

Fig B in S1 Appendix presents PRCCs describing the relationship between individual parameters and total averted costs estimated for each disease. For HIV and TB, the most influential parameters were the duration of hospitalization and the unit cost per hospital bed-day. For malaria, the most influential determinants were the difference in symptomatic malaria cases between scenarios, the number of outpatient visits per malaria case, and the unit cost per outpatient visit.

Table F in S1 Appendix compares the results of our main analysis to two alternative analytic specifications, which allowed PHC unit costs to vary to reflect economies or diseconomies of scale, respectively. When we allowed for a negative relationship between PHC service volume and unit costs (alternative specification 1), overall averted cost estimates were 18% lower than in the main analysis. When we assumed that increased utilization would exceed capacity constraints and require additional investments (alternative specification 2), averted costs were 10% greater than in the main analysis.

**Fig 4:**
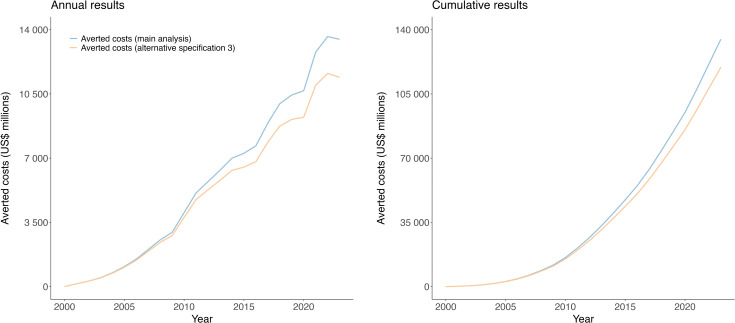
Averted costs due to scale-up of HIV, TB, and malaria services from 2000 to 2023, with and without considering additional survival due to reduced HTM mortality, for actual scenario compared to constant coverage scenario. Cost values represent nominal U.S. dollars. HIV, human immunodeficiency virus; TB, tuberculosis. HTM: HIV, TB, and malaria.

Fig 4 compares results from the main analyses with those from alternative specification 3. This specification accounted for additional PHC utilization in the ***actual scenario*** resulting from greater survival due to averted HTM deaths. Under this specification the total averted PHC costs (summed across all countries, disease, and years) was estimated to be 11% lower than in the main analysis. This difference increased over the time period of the analysis, reaching 15% in 2023.

Fig C in S1 Appendix shows averted costs estimates for main analysis versus alternative specification 3 for each disease. Over the study period, total averted costs were estimated to be 8% lower for HIV, 14% lower for TB, and 7% lower for malaria in the alternative specification (versus main analysis), with differences of 13%, 17%, and 11% estimated for 2023, respectively.

## Discussion

We investigated how investments to scale-up of HIV, TB, and malaria services have influenced PHC in 108 LMICs, specifically through changes in healthcare utilization among persons with untreated HIV, TB, or malaria who are unable to access effective disease-specific care. We estimated substantial reductions in PHC utilization among these persons, with 6.9 billion outpatient visits and 3.9 billion inpatient bed-days averted over the year 2000–2023, as compared to a counterfactual scenario constraining HTM service coverage at year 2000 levels. These reductions have freed up a median of 2.7% hospital beds across the 108 countries, 18.0% across countries in the SSA region, and 16.3% across low-income countries. The total averted PHC utilization translated into US$135 billion in averted costs, representing a median of 1.3% of domestic government health spending across the 108 countries and 6.1% across countries in the SSA region. Additionally, for 13 countries, particularly those with high disease burden and limited health system capacity, the averted PHC utilization exceeds 50% of national hospital bed capacity and 25% of government health spending.

Several previous studies have suggested pathways through which disease-specific investments can enhance health service delivery, improve medical supply chains, reduce the burden of related conditions, and facilitate human resources for health in LMICs [29–32]. The results of this study demonstrate another mechanism through which these investments can contribute to health system strengthening, not usually quantified in other analyses. This work adds to the body of literature documenting the complex consequences of global health initiatives to address specific diseases in resource-limited settings [33–36]. Under the ***constant coverage*** counterfactual scenario, low HTM service coverage allowed for continued disease evolution and spread, and any reductions in disease burden achieved pre-2000 plateaued and reversed. As a consequence, low HTM service coverage was projected to produce an increasing number of individuals with symptomatic HIV, TB, or malaria without effective disease-specific treatment, who would seek primary care when symptoms emerged, and potentially require hospitalization with the development of advanced conditions. In the sensitivity analysis where we examined the ***no coverage*** counterfactual scenario, each disease was projected to expand unchecked, leading to larger numbers of symptomatic individuals seeking PHC in subsequent years. The projected surge in demand for health services under the counterfactual scenarios illustrates the impact that the scale-up of HTM services has had in averting preventable PHC utilization and costs.

Overall, our estimates showed progressive growth in the magnitude of averted PHC utilization and costs over the study period, as HTM services were scaled up and the compounding benefits of transmission reductions were realized. An exception to this pattern was malaria, for which we identified fluctuations in the time trends of estimated outcomes for some countries and years, especially around 2020. These were likely attributable to resurgences of malaria incidence driven by changes in funding mechanisms, COVID-19 pandemic-related disruptions, and multi-year cycles for mass distributions of insecticide-treated bed nets [37–39]. This finding reveals how interruptions in intervention access can have secondary consequences on other healthcare, adding resource and financial burdens to the already strained PHC systems.

In sensitivity analyses, we considered three alternative analytic specifications. In specification 1 and 2, we explored how changes in the cost functions used to calculate PHC costs would affect cost results. The main analysis assumed that PHC unit costs would not change with different utilization levels, such that any change in utilization would produce a proportional change in total costs. When we allowed for unit costs to change with increasing utilization due to economies of scale and diseconomies of scale, the averted costs became 18% lower and 10% higher compared to those estimated in the main analysis, respectively. The balance of these two mechanisms is unknown and will likely differ between countries and according to the magnitude of additional utilization. Nevertheless, it is notable that estimated averted costs were substantial under each of the specifications we explored. In specification 3, we adjusted the main analysis to account for improved survival under actual versus counterfactual scenarios. Over the study period, the estimate of averted costs under this specification was 11% lower than in the main analysis. This reduction reflects the fact that individuals whose deaths were averted due to effective HIV, TB, or malaria services continue to utilize PHC, partially offsetting the reductions in PHC utilization and costs that would otherwise be observed if survival differences were not considered. However, the overall magnitude of results in this specification remains comparable to the main analysis.

This study has several limitations. First, we did not quantify the cost or health impact of the HIV, TB, and malaria programs themselves, as these have been reported elsewhere [19]. Second, our analysis did not consider changes in PHC services beyond utilization. Changes in utilization could also affect service quality, timeliness, and patient satisfaction, but these outcomes were not considered in this study. Third, there is limited evidence on healthcare utilization rates for individuals with untreated HIV, TB, or malaria, increasing the uncertainty of our estimates. Fourth, we used mathematical modeling to estimate the impact of lower HTM service availability. It is possible that empirical approaches, leveraging observed variation in HTM service coverage, could also be used to investigate the impact of lower HTM service availability. Fifth, although we calculated outcomes using country-level epidemiological data, several analytic inputs were only available as global averages or were translated to different country settings using regression adjustments. For this reason, outcomes for individual countries should be interpreted with caution. Sixth, our analysis assumed that PHC access for individuals without HIV, TB, and malaria would be the same across scenarios. For this reason, lower PHC utilization in the ***actual scenario*** is quantified solely as averted costs. However, reductions in utilization for untreated HIV, TB, and malaria could free up PHC services for individuals with other conditions. If so, some of the benefits we quantified as averted PHC costs would instead be realized as additional health benefits. Lastly, some population groups can face systemic access barriers, limiting utilization of both HTM and PHC services. We did not investigate the implications of these correlated patterns of disadvantage. Given these limitations, further research is needed to illuminate the societal and economic impacts of investments to combat major infectious diseases across different LMIC settings, including how supply-side factors play a role in mediating these outcomes.

This analysis provides evidence on how investments to scale up HIV, TB, and malaria services over the start of the 21st century have reduced the burden placed by these diseases on routine health systems. Our findings underscore the positive contribution that sustained support by international donors and domestic governments for HIV, TB, and malaria elimination can make to the broader agenda of promoting health system resilience and efficiency. Under counterfactual scenarios that assumed lower levels of HTM service coverage, we estimated there would have been much greater PHC utilization and costs. While it is unclear how these costs would have been financed, they would have put increasing strain on health systems already experiencing capacity and funding constraints. These consequences would likely be more pronounced for countries of low income, with fragile health systems and high levels of disease burdens, particularly in the SSA region. These avoided capacity and financial strains illustrate how the benefits of HTM investments extend beyond disease-specific outcomes, highlighting the importance of recognizing not only the direct health and economic gains from these investments but also the indirect benefits for primary health systems. These indirect impacts should be explicitly considered when evaluating the full value of global health investments.

## Supporting information

S1 Appendix Supplementary methods, tables, and figures supporting the main analysis. (DOCX)

S1 CHEERS Checklist Consolidated Health Economic Evaluation Reporting Standards (CHEERS) 2022 checklist [14]. (DOCX)

## Abbreviations

ART: antiretroviral therapy
CHEERS: Consolidated Health Economic Evaluation Reporting Standards
HIV: human immunodeficiency virus
HTM: HIV, TB, and malaria
IQR: interquartile range
LMICs: low- and middle-income countries
PHC: primary healthcare
PRCCs: partial rank correlation coefficients
SSA: sub-Saharan Africa
TB: tuberculosis
UI: uncertainty interval
