# Supplementary material for: The benefits of investments to combat HIV, tuberculosis, and malaria for primary healthcare from 2000 to 2023: An economic modeling analysis
Source: PLoS Med. 2026 Apr 8;23(4):e1005036. doi: 10.1371/journal.pmed.1005036 (PMC13061260; doi:10.1371/journal.pmed.1005036)
Supplement: S1 Appendix — (DOCX) [file pmed.1005036.s001.docx]

S1 Appendix

The benefits of investments to combat HIV, tuberculosis and malaria for primary health care from 2000 to 2023: an economic modeling analysis

Table of Contents

[Supplementary methods 1](#_Toc224054686)

[Meta-regression models for HIV input parameters 1](#_Toc224054687)

[Emulator for epidemiological outcomes in additional TB-burden countries 1](#_Toc224054688)

[Regression models for Malaria input parameters 2](#_Toc224054689)

[Table A. List of countries included in the study. 3](#_Toc224054690)

Fig A. Flowchart of outcomes

[Fig A1. Flowchart of outcomes for HIV. 4](#_Toc224054691)

[Fig A2 Flowchart of outcomes for TB. 5](#_Toc224054692)

[Fig A3. Flowchart of outcomes for malaria. 6](#_Toc224054693)

Table B. Summary of model input parameters

[Table B1. Summary of HIV model input parameters. 7](#_Toc224054694)

[Table B2. Summary of TB model input parameters. 8](#_Toc224054695)

[Table B3. Summary of malaria model input parameters. 9](#_Toc224054696)

Table C. Cumulative averted primary health care utilization and associated averted costs due to scale-up services, for actual scenario compared to constant coverage scenario, 2000-23

[Table C1. Cumulative averted primary health care utilization and associated averted costs due to scale-up HIV services, for actual scenario compared to constant coverage scenario, 2000-23. 10](#_Toc224054697)

[Table C2. Cumulative averted primary health care utilization and associated averted costs due to scale-up TB services, for actual scenario compared to constant coverage scenario, 2000-23. 11](#_Toc224054698)

[Table C3. Cumulative averted primary health care utilization and associated averted costs due to scale-up malaria services, for actual scenario compared to constant coverage scenario, 2000-23. 12](#_Toc224054699)

[Table D. Sensitivity analysis – Cumulative averted primary health care utilization and associated averted costs due to scale-up HIV, TB, and malaria services, for actual scenario compared to no coverage scenario, 2000-23. 13](#_Toc224054700)

[Table E. Sensitivity analysis – Estimates of averted inpatient utilization as a percentage of national hospital bed capacity and averted costs as a percentage of domestic government health expenditure, for actual scenario compared to no coverage scenario, 2000-23. 14](#_Toc224054701)

[Fig B. Sensitivity analysis – Partial rank correlation coefficients (PRCCs) for model estimates of averted costs in 2023 for actual scenario compared to constant coverage scenario. 15](#_Toc224054702)

[Table F. Sensitivity analysis – Cumulative averted costs due to scale-up HIV, TB, and malaria services under different unit cost assumptions, for actual scenario compared to constant coverage scenario, 2000-23. 16](#_Toc224054703)

[Fig C. Annual averted costs due to scale-up of HIV, TB, and malaria services from 2000 to 2023, with and without considering additional survival due to reduced HTM mortality, for actual scenario compared to constant coverage scenario. 17](#_Toc224054704)

[References 18](#_Toc224054705)

**Supplementary methods**

## Meta-regression models for HIV input parameters

For each CD4 category stratum *d* (<50, 50–99, 100–199, 200–249, 250–349, 350–499, and ≥500 cells/mm³), we estimated utilization rate ratios (${RR}_{d}^{op\_HIV}$ and ${RR}_{d}^{ip\_HIV}$) for individuals with untreated HIV relative to the general population. These rate ratios were estimated from meta-regression models fit to data extracted from published literature, describing the relative frequency of outpatient visits and hospitalization for individuals with different levels of immunosuppression (references included in Table B1). For both outpatient and inpatient visits, we modeled utilization as a log-linear function of CD4 count and study-level indicator variables, with Gamma-distributed errors.

## Emulator for epidemiological outcomes in additional TB-burden countries

For each scenario, we extracted estimates of epidemiological outcomes for 29 countries from the results of the *Global Portfolio TB Model* [[1](#_ENREF_1)], covering 81% of global TB incidence. To increase the fraction of global TB burden covered by our analysis, we developed a regression-based emulator using the modeled results for 29 countries. The emulator was constructed as two generalized additive models (GAMs), predicting the TB incidence rate and TB treatment coverage, respectively. For the TB incidence rate, we developed a GAM model using a log link function and a Gaussian error distribution. Predictors included smooth terms for the log-transformed TB incidence rate (derived from the WHO burden estimates) [[2](#_ENREF_2)], the percentage of incident TB cases that are HIV-positive [[2](#_ENREF_2)], and calendar year. The number of smooth terms for modelled predictors was chosen to minimize root mean square prediction error based on leave-one-out cross-validation. For the *actual scenario*, the annual number of new TB cases was calculated by multiplying the TB incidence rate (as predicted by the regression model) by annual population. For the *constant coverage scenario* and *the no coverage scenario*, we refit the emulation model using the ratio of annual TB cases in each counterfactual scenario relative to the number of annual TB cases in the *actual scenario* as the predicted variable. We set year 2000 as the base year, for which outcomes were assumed to match those of the *actual scenario*. The final estimates of new TB cases in counterfactual scenarios were then imputed as the number of new TB cases in the *actual scenario* multiplied by the ratios estimated from the regression model.

To model treatment coverage, we fit a GAM model with a logit link function and a Gaussian error distribution. The model included smooth terms for logit-transformed treatment coverage [[2](#_ENREF_2)], the percentage of incident TB cases that are HIV-positive [[2](#_ENREF_2)], and calendar year. The predicted variable for the *actual scenario* was the TB treatment coverage. The predicted variable for the *constant coverage scenario* was the ratio of TB treatment coverage in this scenario compared to the *actual scenario*. We set year 2000 as the base year, and calculated treatment coverage in the *constant coverage scenario* as treatment coverage for the *actual scenario* multiplied by the ratios estimated from the regression model. Treatment coverage for the *no coverage scenario* was set to zero over the study period. Using this emulator, we predicted epidemiological results for each scenario for an additional set of 22 countries not included in the original modeled results. These countries were chosen based on their inclusion in WHO lists of high-burden countries (for TB, TB-HIV, or MDR-TB) [[3](#_ENREF_3)] and covered 11% of global TB incidence.

## Regression models for Malaria input parameters

For each country and calendar year, we calculated the proportion of individuals with symptomatic malaria that seek care ($P_{c, y}^{care}$ in Equation 5 in main text), based on a regression model fit to country-level data on treatment coverage as collected through Demographic and Health (DHS) Surveys [[4](#_ENREF_4)]. This model was constructed as a generalized linear mixed model with a logit link function and a Gaussian error distribution, for which we included random effects for country, a linear term for time (centered on year 2000), and a covariate for the universal health coverage index [[5](#_ENREF_5)]. Using the same regression model structure we developed models to estimate the number of hospitalizations per uncomplicated malaria case ($P_{c}^{unc}$) and per severe case ($P_{c}^{sev})$ by country, as used in Equation 6 in the main text.

# **Table A. List of countries included in the study.**

| **Disease** | **Countries** |
| --- | --- |
| HIV  (n=108) | Afghanistan, Angola, Armenia, Azerbaijan, Burundi, Benin, Burkina Faso, Bangladesh, Belarus, Belize, Bolivia, Brazil, Bhutan, Botswana, Central African Republic, China, Côte d’Ivoire, Cameroon, Democratic Republic of the Congo, Republic of the Congo, Colombia, Comoros, Cabo Verde, Costa Rica, Cuba, Djibouti, Dominican Republic, Algeria, Ecuador, Egypt, Eritrea, Ethiopia, Fiji, Gabon, Georgia, Ghana, Guinea, The Gambia, Guinea-Bissau, Equatorial Guinea, Guatemala, Honduras, Haiti, Indonesia, India, Iran, Jamaica, Kazakhstan, Kenya, Kyrgyzstan, Cambodia, Lao People’s Democratic Republic, Lebanon, Liberia, Sri Lanka, Lesotho, Morocco, Moldova, Madagascar, Maldives, Mexico, North Macedonia, Mali, Myanmar, Montenegro, Mongolia, Mozambique, Mauritania, Mauritius, Malawi, Malaysia, Namibia, Niger, Nigeria, Nicaragua, Nepal, Pakistan, Peru, Philippines, Papua New Guinea, Paraguay, Rwanda, Sudan, Senegal, Sierra Leone, El Salvador, Somalia, Serbia, South Sudan, São Tomé and Príncipe, Suriname, Eswatini, Syria, Chad, Togo, Thailand, Tajikistan, Timor-Leste, Tunisia, United Republic of Tanzania, Uganda, Ukraine, Uzbekistan, Vietnam, Yemen, South Africa, Zambia, Zimbabwe |
| TB  (n=51) | Afghanistan, Angola, Azerbaijan, Belarus, Bangladesh, Botswana, Brazil, Cambodia, Cameroon, Central African Republic, China, Democratic Republic of the Congo, Republic of the Congo, Ethiopia, Gabon, Ghana, Guinea, Guinea-Bissau, India, Indonesia, Kazakhstan, Kenya, Kyrgyzstan, Liberia, Lesotho, Madagascar, Malawi, Moldova, Mongolia, Mozambique, Myanmar, Namibia, Nepal, Nigeria, Pakistan, Papua New Guinea, Peru, Philippines, Sierra Leone, Somalia, Eswatini, Tajikistan, Thailand, United Republic of Tanzania, Uganda, Ukraine, Uzbekistan, Vietnam, South Africa, Zambia, Zimbabwe |
| Malaria  (n=55) | Afghanistan, Angola, Burundi, Benin, Burkina Faso, Bangladesh, Central African Republic, Côte d’Ivoire, Cameroon, Democratic Republic of the Congo, Republic of the Congo, Comoros, Djibouti, Eritrea, Ethiopia, Ghana, Guinea, The Gambia, Guinea-Bissau, Guatemala, Honduras, Haiti, Indonesia, India, Kenya, Cambodia, Lao People’s Democratic Republic, Liberia, Madagascar, Mali, Myanmar, Mozambique, Mauritania, Malawi, Namibia, Niger, Nigeria, Nicaragua, Pakistan, Philippines, Papua New Guinea, Rwanda, Sudan, Senegal, Sierra Leone, Somalia, South Sudan, Chad, Togo, Thailand, United Republic of Tanzania, Uganda, Vietnam, Zambia, Zimbabwe |

HIV: human immunodeficiency virus; TB: tuberculosis.

## **Fig A1. Flowchart of outcomes for HIV.**





HIV: human immunodeficiency virus; TB: tuberculosis.

## **Fig A2 Flowchart of outcomes for TB.**





HIV: human immunodeficiency virus; TB: tuberculosis.

## **Fig A3. Flowchart of outcomes for malaria.**





HIV: human immunodeficiency virus; TB: tuberculosis.

## **Table B1. Summary of HIV model input parameters.**

| Input parameter | Mean value  (95% uncertainty interval) ^‡^ | Distribution ^†^ | Reference |
| --- | --- | --- | --- |
| Number of individuals with HIV not on ART | Stratified by country, year, age group, CD4 cell count category, and scenario | Gamma | Epidemiological models [[6](#_ENREF_6), [7](#_ENREF_7)] |
| Population | Stratified by country and year | Fixed value |  |
| Annual number of outpatient visits per disease-negative individual | Stratified by country, year, and age group | Gamma | IHME healthcare utilization estimates [[8](#_ENREF_8)] |
| Annual number of hospitalizations per disease-negative individual | Stratified by country, year, and age group | Gamma |  |
| Rate ratio of outpatient visits for individuals with HIV not on ART, relative to HIV-negative individuals |  | Gamma | Estimated from meta-regression model fit to published values [[9-13](#_ENREF_9)] |
| CD4 count 0-49 cells/mm^3^ | 4.4 (1.9, 9.0) |  |  |
| CD4 count 50-99 cells/mm^3^ | 4.0 (1.8, 7.9) |  |  |
| CD4 count 100-199 cells/mm^3^ | 3.5 (1.7, 6.4) |  |  |
| CD4 count 200-249 cells/mm^3^ | 3.0 (1.6, 5.3) |  |  |
| CD4 count 250-349 cells/mm^3^ | 2.7 (1.5, 4.3) |  |  |
| CD4 count 350-499 cells/mm^3^ | 2.1 (1.4, 3.1) |  |  |
| CD4 count ≥ 500 cells/mm^3^ | 1.5 (1.2, 1.8) |  |  |
| Rate ratio of hospitalization for individuals with HIV not on ART, relative to HIV-negative individuals |  | Gamma | Estimated from meta-regression model fit to published values [[9-12](#_ENREF_9), [14](#_ENREF_14)] |
| CD4 counts 0-49 cells/mm^3^ | 42.1 (13.9, 99.4) |  |  |
| CD4 counts 50-99 cells/mm^3^ | 33.3 (11.8, 75.2) |  |  |
| CD4 counts 100-199 cells/mm^3^ | 23.5 (9.3, 49.5) |  |  |
| CD4 counts 200-249 cells/mm^3^ | 16.6 (7.3, 32.6) |  |  |
| CD4 counts 250-349 cells/mm^3^ | 11.8 (5.8, 21.5) |  |  |
| CD4 counts 350-499 cells/mm^3^ | 6.7 (3.9, 10.7) |  |  |
| CD4 counts ≥ 500 cells/mm^3^ | 2.7 (2.0, 3.5) |  |  |
| Mean duration of hospital stays, days | 6.6 (4.0, 13.0) | Gamma | Published estimates [[13](#_ENREF_13), [14](#_ENREF_14)] |
| Cost per outpatient visit ^§^ | Stratified by country and year | Gamma | WHO-CHOICE estimates [[15](#_ENREF_15)] |
| Cost per inpatient bed-day ^§^ | Stratified by country and year | Gamma | WHO-CHOICE estimates [[15](#_ENREF_15)] |

^‡^ We assumed a 95% interval equivalent to +/- 50% of the point estimate for parameters for which measures of uncertainty were not available.

^†^ To create prior distributions for probabilistic sensitivity analysis, we specified distributions matching the mean value and interval width for each input parameter.

^§^ Same unit cost (cost per outpatient visit, cost per inpatient bed-day) values were applied to all three diseases.

HIV: human immunodeficiency virus.

## **Table B2. Summary of TB model input parameters.**

| Input parameter | Mean value  (95% uncertainty interval) ^‡^ | Distribution ^†^ | Reference |
| --- | --- | --- | --- |
| Number of new TB cases | Stratified by country, year, and scenario | Gamma | Epidemiological models [[6](#_ENREF_6), [7](#_ENREF_7)] |
| Number of new TB cases receiving effective TB treatment | Stratified by country, year, and scenario | Gamma |  |
| Number of new TB cases, additional countries | Stratified by country, year, and scenario | Multivariate normal for logged outcome | Imputed using model emulator |
| Treatment coverage, additional countries | Stratified by country, year, and scenario | Multivariate normal for log-odds of outcome |  |
| Mean outpatient visits per disease-negative individual | Stratified by country and year | Gamma | IHME healthcare utilization estimates [8] |
| Mean duration of pre-diagnostic period for symptomatic TB cases receiving effective TB treatment, months | 1.0 (0.5, 1.5) | Gamma | Published estimates [16, 17] |
| Rate ratio of outpatient visits per TB case not receiving effective TB treatment, relative to TB-negative case | 6.23 (4.49, 11.47) | Gamma | Published estimates [18-26] |
| Mean duration of symptomatic TB cases not receiving effective TB treatment, months | 6.0 (3.0, 9.0) | Gamma | Published estimates of untreated disease duration [27, 28], adjusted for symptom prevalence [29] ᵟ |
| Case fatality rate for untreated TB | 0.43 (0.28, 0.53) | Beta | WHO Global TB Program Methods Supplement [30] |
| Mean duration of hospital stays, days | 14.0 (7.0, 21.0) | Gamma | WHO TB patient cost surveys 2015-2021 [31] |
| Cost per outpatient visit ^§^ | Stratified by country and year | Gamma | WHO-CHOICE estimates [15] |
| Cost per inpatient bed-day ^§^ | Stratified by country and year | Gamma | WHO-CHOICE estimates [15] |

^‡^ We assumed a 95% interval equivalent to +/- 50% of the point estimate for parameters where measures of uncertainty were not available.

^†^ To create prior distributions for probabilistic sensitivity analysis, we specified distributions matching the mean value and interval width for each input parameter.

ᵟ There is substantial uncertainty about the duration of untreated TB (estimates range from 1.6 to 5.4 years) and the prevalence of symptoms (estimates range from 36 to 80%). Our estimate (mean 6 months) represents a conservative value based on available evidence.

^§^ Same unit cost (cost per outpatient visit, cost per inpatient bed-day) values were applied to all three diseases.

TB: tuberculosis.

## **Table B3. Summary of malaria model input parameters.**

| Input parameter | Mean value  (95% uncertainty interval) ^‡^ | Distribution ^†^ | Reference |
| --- | --- | --- | --- |
| Number of symptomatic malaria cases | Stratified by country, year, and scenario | Gamma | Epidemiological models [[6](#_ENREF_6), [7](#_ENREF_7)] |
| Treatment coverage | Stratified by country, year, and scenario | Fixed value |  |
| Number of malaria deaths | Stratified by country, year and scenario | Gamma |  |
| Number of hospitalized malaria cases receiving effective malaria care | Stratified by country, year and scenario | Fixed value |  |
| Outpatient visits per malaria case | 1.0 (0.5, 1.5) | Gamma | Estimated from published estimates [4] |
| Proportion of individuals with malaria seeking care | Stratified by country and year | Multivariate normal for log-odds of outcome | Estimated from regression model fit to published values [4] |
| Hospitalizations per malaria case | Stratified by country | Multivariate normal for logged outcome | Estimated from regression model fit to published values [5, 33] |
| Hospitalizations per malaria death | Stratified by country | Multivariate normal for logged of outcome | Estimated from regression model fit to published values [5, 33] |
| Inpatient bed-days per hospitalized malaria case | 3.0 (1.5, 4.5) | Gamma | Published estimate [34] |
| Cost per outpatient visit ^§^ | Stratified by country and year | Gamma | WHO-CHOICE estimates [15] |
| Cost per inpatient bed-day ^§^ | Stratified by country and year | Gamma | WHO-CHOICE estimates [15] |

^‡^ We assumed a 95% interval equivalent to +/- 50% of the point estimate for parameters where measures of uncertainty were not available.

^†^ To create prior distributions for probabilistic sensitivity analysis, we specified distributions matching the mean value and interval width for each input parameter.

^§^ Same unit cost (cost per outpatient visit, cost per inpatient bed-day) values were applied to all three diseases.

## **Table C1. Cumulative averted primary health care utilization and associated averted costs due to scale-up HIV services, for actual scenario compared to constant coverage scenario, 2000-23.**

|  | Averted utilization, millions | | | | Averted costs (US$), millions | | |
| --- | --- | --- | --- | --- | --- | --- | --- |
|  | Outpatient  visits | | Inpatient  bed-days | | Outpatient | Inpatient | Total |
| Total | 1188  (757, 1734) | 1352  (516, 2710) | | 4049  (1930, 7688) | | 50130  (14685, 124045) | 54179  (17981, 129624) |
| World Bank region | | | | | | | |
| East Asia and Pacific | 111  (67, 163) | 113  (40, 222) | | 566  (157, 1432) | | 5356  (1251,13285) | 5921  (1730, 13845) |
| Europe and Central Asia | 27  (17, 40) | 33  (13, 64) | | 129  (53, 256) | | 1260  (413, 2788) | 1389  (540, 2957) |
| Latin America and Caribbean | 89  (56, 126) | 73  (28, 149) | | 453  (188, 903) | | 2743  (885, 6328) | 3196  (1268, 6827) |
| Middle East and North Africa | 4.0  (2.5, 5.8) | 3.4  (1.3, 6.8) | | 25  (10, 52) | | 165  (54, 379) | 190  (73, 413) |
| South Asia | 54  (28, 88) | 47  (16, 101) | | 91  (12, 257) | | 482  (109, 1334) | 573  (165, 1408) |
| Sub-Saharan Africa | 903  (577, 1327) | 1083  (412, 2175) | | 2786  (1030, 6563) | | 40124  (9543, 111753) | 42910  (12845, 113225) |
| Income classification | | | | | | | |
| Low income | 273  (169, 402) | 183  (71, 364) | | 215  (95, 423) | | 619  (211, 1353) | 834  (392, 1613) |
| Lower middle income | 460  (286, 674) | 424  (166, 874) | | 749  (396, 1267) | | 3787  (1435, 7897) | 4536  (2185, 8824) |
| Upper middle income | 455  (282, 690) | 744  (256, 1627) | | 3085  (1169, 6726) | | 45724  (11950, 119344) | 48809  (14829, 121741) |
| Time period | | | | | | | |
| 2000-2009 | 79  (45, 124) | 99  (36, 202) | | 222  (100, 411) | | 3081  (836, 8297) | 3303  (1064, 8567) |
| 2010-2019 | 627  (401, 923) | 735  (274, 1470) | | 2199  (1009, 4368) | | 28484  (7992, 71657) | 30683  (9881, 74618) |
| 2020-2023 | 482  (310, 692) | 518  (203, 1018) | | 1628  (791, 3029) | | 18565  (5550, 45059) | 20193  (7065, 46938) |

Values in parentheses represent 95% uncertainty intervals. Cost values represent nominal U.S. dollars. Actual scenario represents the observed scale-up of HIV services over the study period. Constant coverage scenario represents a counterfactual with coverage of HIV services held constant at the year 2000 levels for each country over the study period.

HIV: human immunodeficiency virus.

## **Table C2. Cumulative averted primary health care utilization and associated averted costs due to scale-up TB services, for actual scenario compared to constant coverage scenario, 2000-23.**

|  | Averted utilization, millions | | Averted costs (US$), millions | | |
| --- | --- | --- | --- | --- | --- |
|  | Outpatient  visits | Inpatient  bed-days | Outpatient | Inpatient | Total |
| Total | 3289  (1090, 6658) | 2146  (1177, 3526) | 13394  (3296, 38136) | 61336  (24752, 125018) | 74730  (34373, 143816) |
| World Bank region | | | | | |
| East Asia and Pacific | 1676  (557, 3449) | 1005  (554, 1695) | 9688  (1395, 33106) | 43448  (13614, 99613) | 53136  (19758, 112956) |
| Europe and Central Asia | 179  (60, 362) | 62  (34, 102) | 776  (214, 1982) | 2152  (937, 4108) | 2928  (1496, 5083) |
| Latin America and Caribbean | 156  (49, 335) | 83  (42, 148) | 367  (57, 1118) | 1608  (480, 3707) | 1975  (661, 4169) |
| Middle East and North Africa | - | - | - | - | - |
| South Asia | 619  (212, 1285) | 492  (267, 823) | 970  (175, 2763) | 4847  (1601, 10823) | 5816  (2299, 12251) |
| Sub-Saharan Africa | 658  (226, 1348) | 505  (276, 843) | 1592  (428, 4041) | 9282  (3725, 18910) | 10874  (5102, 20844) |
| Income classification | | | | | |
| Low income | 315  (106, 651) | 234  (128, 388) | 236  (66, 607) | 729  (328, 1433) | 965  (495, 1790) |
| Lower middle income | 1133  (389, 2288) | 821  (446, 1369) | 2181  (660, 5113) | 9609  (4181, 18473) | 11790  (5925, 21211) |
| Upper middle income | 1841  (614, 3756) | 1091  (595, 1948) | 10977  (2066, 34811) | 50998  (18206, 110109) | 61975  (26013, 125088) |
| Time period | | | | | |
| 2000-2009 | 392  (129, 801) | 342  (191, 561) | 967  (276, 2425) | 6755  (2812, 13399) | 7722  (3667, 14505) |
| 2010-2019 | 1672  (564, 3400) | 1087  (603, 1788) | 6768  (1695, 19244) | 31492  (12788, 63884) | 38261  (17720, 72101) |
| 2020-2023 | 1225  (411, 2509) | 717  (390, 1190) | 5659  (1306, 16680) | 23089  (8719, 48807) | 28747  (12965, 57032) |

Values in parentheses represent 95% uncertainty intervals. Cost values represent nominal U.S. dollars. Actual scenario represents the observed scale-up of TB services over the study period. Constant coverage scenario represents a counterfactual with coverage of TB services held constant at the year 2000 levels for each country over the study period.

TB: tuberculosis.

## **Table C3. Cumulative averted primary health care utilization and associated averted costs due to scale-up malaria services, for actual scenario compared to constant coverage scenario, 2000-23.**

|  | Averted utilization, millions | | Averted costs (US$), millions | | |
| --- | --- | --- | --- | --- | --- |
|  | Outpatient  visits | Inpatient  bed-days | Outpatient | Inpatient | Total |
| Total | 2435  (1369, 3803) | 395  (184, 767) | 3065  (1455, 5424) | 2813  (1188, 6048) | 5878  (3357, 9531) |
| World Bank region | | | | | |
| East Asia and Pacific | 39  (20, 65) | 8.1  (3.5, 16.8) | 96  (28, 250) | 101  (39, 221) | 197  (91, 376) |
| Europe and Central Asia | - | - | - | - | - |
| Latin America and Caribbean | 1.1  (0.6, 1.9) | 0.4  (0.1, 1.4) | 2.7  (0.9, 5.9) | 6.2  (0.9, 23.2) | 8.9  (2.6, 26.3) |
| Middle East and North Africa | 1.3  (0.6, 2.3) | 0.3  (0.1, 1.0) | 3.0  (0.3, 9.1) | 4.3  (0.8, 15.4) | 7.3  (1.9, 19.8) |
| South Asia | 115  (55, 210) | 56  (10, 165) | 181  (25, 508) | 558  (65, 2066) | 739  (159, 2237) |
| Sub-Saharan Africa | 2278  (1275, 3582) | 330  (159, 591) | 2783  (1305, 5008) | 2144  (965, 4370) | 4926  (2815, 7816) |
| Income classification | | | | | |
| Low income | 1326  (730, 2089) | 207  (94, 387) | 1198  (550, 2210) | 1089  (404, 2779) | 2287  (1218, 4194) |
| Lower middle income | 1104  (598, 1779) | 186  (84, 384) | 1854  (754, 3832) | 1691  (688, 3709) | 3545  (1928, 6119) |
| Upper middle income | 5.1  (2.5, 9.1) | 2.1  (0.6, 4.7) | 13  (2, 39) | 33  (5, 97) | 46  (14, 116) |
| Time period | | | | | |
| 2000-2009 | 393  (222, 618) | 62  (30, 124) | 406  (189, 756) | 364  (152, 813) | 770  (438, 1306) |
| 2010-2019 | 1404  (794, 2205) | 245  (112, 475) | 1776  (848, 3138) | 1728  (721, 3818) | 3503  (2003, 5698) |
| 2020-2023 | 637  (353, 1002) | 88  (40, 164) | 883  (415, 1587) | 721  (303, 1574) | 1604  (899, 2549) |

Values in parentheses represent 95% uncertainty intervals. Cost values represent nominal U.S. dollars. Actual scenario represents the observed scale-up of malaria services over the study period. Constant coverage scenario represents a counterfactual with coverage of malaria services held constant at the year 2000 levels for each country over the study period.

# **Table D. Sensitivity analysis – Cumulative averted primary health care utilization and associated averted costs due to scale-up HIV, TB, and malaria services, for actual scenario compared to no coverage scenario, 2000-23.**

|  | Averted utilization, millions | | Averted costs (US$), millions | | |
| --- | --- | --- | --- | --- | --- |
|  | Outpatient  visits | Inpatient  bed-days | Outpatient | Inpatient | Total |
| Total | 18232  (10380, 29788) | 10109  (6292, 15551) | 57819  (22074, 140122) | 299381  (137892, 582523) | 357200  (183702, 650937) |
| Disease | | | | | |
| HIV | 1580  (998, 2310) | 2032  (772, 4178) | 6803  (2523, 16035) | 93798  (22609, 252183) | 100601  (29610, 262900) |
| TB | 11113  (3734, 22383) | 7296  (3942, 12178) | 43877  (10990, 125346) | 199705  (76701, 419880) | 243582  (113249, 475521) |
| Malaria | 5540  (3083, 8727) | 781  (380, 1511) | 7139  (3343, 12971) | 5878  (2461, 13036) | 13017  (7549, 21706) |
| World Bank region | | | | | |
| East Asia and Pacific | 5832  (2065, 11886) | 3445  (1868, 5847) | 32754  (5156, 109195) | 148182  (48180, 346274) | 180935  (69613, 391321) |
| Europe and Central Asia | 546  (196, 1084) | 219  (130, 351) | 2501  (725, 6073) | 8132  (3939, 15193) | 10633  (5688, 17912) |
| Latin America and Caribbean | 609  (271, 1146) | 356  (207, 578) | 1738  (512, 4427) | 8454  (3530, 17332) | 10191  (4819, 19346) |
| Middle East and North Africa | 6.7  (4.5, 9.3) | 4.8  (2.0, 9.2) | 33  (14, 64) | 198  (67, 448) | 231  (94, 479) |
| South Asia | 3584  (1645, 6496) | 2417  (1406, 3873) | 5681  (1140, 15860) | 24387  (8150, 54545) | 30067  (11559, 61343) |
| Sub-Saharan Africa | 7655  (5002, 10842) | 3666  (2268, 5935) | 15114  (7433, 29211) | 110029  (35841, 272530) | 125143  (50422, 291688) |
| Income classification | | | | | |
| Low income | 3552  (2191, 5227) | 1179  (792, 1701) | 2934  (1474, 4826) | 4286  (2479, 7061) | 7220  (4743, 10864) |
| Lower middle income | 7907  (4503, 12749) | 4064  (2552, 6316) | 13816  (6309, 27506) | 44164  (21855, 84445) | 57980  (32978, 99161) |
| Upper middle income | 6773  (2796, 13216) | 4866  (2853, 7846) | 41068  (10049, 115039) | 250932  (101827, 521197) | 292000  (133091, 570762) |
| Time period | | | | | |
| 2000-2009 | 2611  (1583, 3916) | 1314  (850, 1977) | 4513  (2282, 8495) | 27591  (12851, 51008) | 32105  (17241, 56960) |
| 2010-2019 | 8980  (5150, 14581) | 5038  (3165, 7669) | 28205  (11246, 66476) | 152425  (70172, 294358) | 180630  (93027, 334233) |
| 2020-2023 | 6642  (3442, 11663) | 3757  (2290, 5877) | 25100  (8390, 66581) | 119365  (54105, 229895) | 144465  (73930, 266971) |

Values in parentheses represent 95% uncertainty intervals. Cost values represent nominal U.S. dollars. Actual scenario represents the observed scale-up of HIV, TB, and malaria services over the study period. No coverage scenario represents a counterfactual with zero coverage of HIV, TB, and malaria services from year 2000 onward for each country over the study period. HIV: human immunodeficiency virus; TB: tuberculosis.

# **Table E. Sensitivity analysis – Estimates of averted inpatient utilization as a percentage of national hospital bed capacity and averted costs as a percentage of domestic government health expenditure, for actual scenario compared to no coverage scenario, 2000-23.**

|  | Number of countries included in analysis | Averted inpatient bed-days  (% of national hospital bed capacity) | | Averted costs (% of domestic government health spending) | |
| --- | --- | --- | --- | --- | --- |
|  |  | Whole period (2000-23) | Final year (2023) | Whole period (2000-23) | Final year (2023) |
| Total | 108 | 6.1 (0.7, 34.3) | 11.0 (1.2, 48.8) | 3.0 (0.1, 8.7) | 3.9 (0.2, 16.1) |
| Disease | | | | | |
| HIV | 108 | 0.7 (0.2, 3.0) | 1.4 (0.4, 5.6) | 0.2 (0.05, 0.8) | 0.3 (0.07, 1.5) |
| TB | 51 | 16.6 (6.1, 30.2) | 26.9 (11.5, 57.6) | 4.5 (2.4, 7.2) | 7.9 (3.5, 14.0) |
| Malaria | 55 | 6.0 (1.5, 11.7) | 5.5 (1.0, 11.9) | 2.9 (0.7, 5.4) | 3.3 (0.7, 6.3) |
| World Bank region | | | | | |
| East Asia and Pacific | 13 | 6.8 (0.8, 13.1) | 11.6 (1.1, 18.7) | 2.4 (0.6, 3.8) | 3.2 (1.0, 4.6) |
| Europe and Central Asia | 13 | 1.9 (0.5, 3.9) | 6.6 (1.2, 9.9) | 2.1 (0.3, 5.3) | 3.5 (0.3, 9.0) |
| Latin America and Caribbean | 18 | 0.8 (0.5, 1.1) | 1.5 (0.9, 2.2) | 0.1 (0.1, 0.2) | 0.2 (0.09, 0.3) |
| Middle East and North Africa | 9 | 0.1 (0.04, 0.14) | 0.2 (0.1, 0.5) | 0.04 (0.02, 0.06) | 0.1 (0.05, 0.2) |
| South Asia | 8 | 12.9 (0.1, 26.5) | 23.4 (0.3, 55.0) | 3.4 (0.04, 6.1) | 5.3 (0.06, 10.0) |
| Sub-Saharan Africa | 47 | 34.7 (11.2, 58.1) | 44.7 (11.8, 96.4) | 9.2 (4.4, 16.7) | 17.2 (5.4, 31.4) |
| Income classification | | | | | |
| Low income | 25 | 35.1 (11.6, 53.3) | 44.7 (11.9, 97.7) | 6.5 (3.6, 16.2) | 9.8 (5.1, 25.1) |
| Lower middle income | 44 | 9.2 (1.0, 34.3) | 13.9 (1.3, 55.7) | 3.9 (0.3, 8.3) | 4.4 (0.6, 16.2) |
| Upper middle income | 39 | 1.1 (0.4, 4.6) | 2.3 (0.6, 10.2) | 0.2 (0.09, 2.5) | 0.3 (0.09, 3.5) |

Point estimates represent median across modeled countries. Values in parentheses represent interquartile range (25^th^ and 75^th^ percentiles) of country-level values. HIV: human immunodeficiency virus; TB: tuberculosis.

# **Fig B. Sensitivity analysis – Partial rank correlation coefficients (PRCCs) for model estimates of averted costs in 2023 for actual scenario compared to constant coverage scenario.**





PRCCs estimated for each country individually based on posterior distribution of model parameters. Values (points) indicate median PRCC across countries. Dark lines represent interquartile range of country-level PRCC values. Light lines represent 10^th^–90^th^ percentiles of country-level PRCC values. HIV: human immunodeficiency virus; TB: tuberculosis.

# **Table F. Sensitivity analysis – Cumulative averted costs due to scale-up HIV, TB, and malaria services under different unit cost assumptions, for actual scenario compared to constant coverage scenario, 2000-23.**

|  | Averted costs (US$), millions | | |
| --- | --- | --- | --- |
|  | Main analysis | Alternative specification 1: economies of scale | Alternative specification 2: exceed capacity constraints |
| Total | 134787  (71025, 250281) | 110282  (51981, 210543) | 148728  (77287, 278326) |
| World Bank region | | | |
| East Asia and Pacific | 59254  (22358, 122284) | 48020  (4842, 100504) | 61937  (23302, 128197) |
| Europe and Central Asia | 4317  (2361, 7169) | 3622  (1914, 6028) | 4483  (2463, 7417) |
| Latin America and Caribbean | 580  (2472, 9584) | 4266  (1669, 8072) | 5350  (2553, 10019) |
| Middle East and North Africa | 197  (80, 421) | 163  (61, 348) | 198  (80, 422) |
| South Asia | 7128  (2846, 14646) | 6028  (2474, 12271) | 7424  (2960, 15267) |
| Sub-Saharan Africa | 58710  (25214, 135051) | 48184  (20297, 109528) | 69336  (28954, 163739) |
| Income classification | | | |
| Low income | 4085  (2661, 6206) | 3403  (2146, 5282) | 4520  (2904, 6949) |
| Lower middle income | 19871  (12599, 31425) | 16673  (10089, 26014) | 21401  (13657, 33942) |
| Upper middle income | 110831  (51968, 221045) | 90207  (35195, 180661) | 122807  (56953, 245327) |
| Time period | | | |
| 2000-2009 | 11795  (6522, 20529) | 9234  (5040, 16416) | 12517  (6879, 22130) |
| 2010-2019 | 72447  (38436, 134776) | 60493  (31191, 112182) | 80213  (41671, 150475) |
| 2020-2023 | 50545  (26424, 94321) | 42212  (21288, 79973) | 55997  (28837, 106000) |

Values in parentheses represent 95% uncertainty intervals. Cost values represent nominal U.S. dollars. Actual scenario represents the observed scale-up of TB services over the study period. Constant coverage scenario represents a counterfactual with coverage of TB services held constant at the year 2000 levels for each country over the study period. HIV: human immunodeficiency virus; TB: tuberculosis.

# **Fig C. Annual averted costs due to scale-up of HIV, TB, and malaria services from 2000 to 2023, with and without considering additional survival due to reduced HTM mortality, for actual scenario compared to constant coverage scenario.**





Cost values represent nominal U.S. dollars. HIV: human immunodeficiency virus; TB: tuberculosis.

# **References**

1. Pretorius C. GlobalPortfolioModel 2025 [cited 2025 Mar 21]. Available from: <https://github.com/CarelPretorius/GlobalTBPortfolioModel>

2. World Health Organization. WHO TB burden estimates 2024 [cited 2024 Nov 8]. Available from: <https://www.who.int/teams/global-tuberculosis-programme/data>.

3. World Health Organization. Global tuberculosis report 2024. Geneva, Switzerland: 2024.

4. ICF. The DHS Program STATcompiler: Funded by USAID; 2015 [cited 2024 Nov 26]. Available from: <http://www.statcompiler.com>.

5. World Health Organization. UHC Service Coverage Index (SDG 3.8.1) 2023 [cited 2024 Dec 10]. Available from: <https://www.who.int/data/gho/data/indicators/indicator-details/GHO/uhc-index-of-service-coverage>.

6. The Global Fund. Investment Case Eighth Replenishment 2025. 2025.

7. Hallett TB, Menzies NA, Resch S, Pretorius C, Stover J, Su JS, et al. The case for optimal investment in combating HIV, tuberculosis, and malaria: a global modelling study. The Lancet. 2025;406(10500):261–70. doi: 10.1016/S0140-6736(25)00831-1.

8. Institute for Health Metrics and Evaluation (IHME). Global Inpatient and Outpatient Health Care Utilization, Unit Costs, and Costs and Services Needed to Achieve Universal Health Coverage 1990-2016 2018 [updated 2022; cited 2024 Mar 31]. Available from: <https://ghdx.healthdata.org/record/ihme-data/UHC-cost-and-services-2016>.

9. Badri M, Cleary S, Maartens G, Pitt J, Bekker LG, Orrell C, et al. When to initiate highly active antiretroviral therapy in sub-Saharan Africa? A South African cost-effectiveness study. Antivir Ther. 2006;11(1):63–72. PubMed PMID: 16518961.

10. Teerawattananon Y, Hanshaoworakul W, Russell S, Tangcharoensathien V, Jiamton S. Targeting antiretroviral therapy: lessons from a longitudinal study of morbidity and treatment in relation to CD4 count in Thailand. Asia Pac J Public Health. 2006;18(1):39–48. doi: 10.1177/10105395060180010701. PubMed PMID: 16629437.

11. Harling G, Orrell C, Wood R. Healthcare utilization of patients accessing an African national treatment program. BMC Health Serv Res. 2007;7:80. Epub 20070607. doi: 10.1186/1472-6963-7-80. PubMed PMID: 17555564; PubMed Central PMCID: PMCPMC1899174.

12. Cleary SM, McIntyre D, Boulle AM. The cost-effectiveness of antiretroviral treatment in Khayelitsha, South Africa--a primary data analysis. Cost Eff Resour Alloc. 2006;4:20. Epub 20061206. doi: 10.1186/1478-7547-4-20. PubMed PMID: 17147833; PubMed Central PMCID: PMCPMC1770938.

13. Siregar AY, Tromp N, Komarudin D, Wisaksana R, van Crevel R, van der Ven A, et al. Costs of HIV/AIDS treatment in Indonesia by time of treatment and stage of disease. BMC Health Serv Res. 2015;15:440. Epub 20150930. doi: 10.1186/s12913-015-1098-3. PubMed PMID: 26424195; PubMed Central PMCID: PMCPMC4590258.

14. Meyer-Rath G, Brennan AT, Fox MP, Modisenyane T, Tshabangu N, Mohapi L, et al. Rates and cost of hospitalization before and after initiation of antiretroviral therapy in urban and rural settings in South Africa. J Acquir Immune Defic Syndr. 2013;62(3):322–8. doi: 10.1097/QAI.0b013e31827e8785. PubMed PMID: 23187948; PubMed Central PMCID: PMCPMC3625673.

15. Stenberg K, Lauer JA, Gkountouras G, Fitzpatrick C, Stanciole A. Econometric estimation of WHO-CHOICE country-specific costs for inpatient and outpatient health service delivery. Cost Effectiveness and Resource Allocation. 2018;16(1):11. doi: 10.1186/s12962-018-0095-x.

16. Storla DG, Yimer S, Bjune GA. A systematic review of delay in the diagnosis and treatment of tuberculosis. BMC public health. 2008;8(1):1–9.

17. Teo AKJ, Singh SR, Prem K, Hsu LY, Yi S. Duration and determinants of delayed tuberculosis diagnosis and treatment in high-burden countries: a mixed-methods systematic review and meta-analysis. Respir Res. 2021;22(1):251. Epub 20210923. doi: 10.1186/s12931-021-01841-6. PubMed PMID: 34556113; PubMed Central PMCID: PMCPMC8459488.

18. Kaswa M, Minga G, Nkiere N, Mingiedi B, Eloko G, Nguhiu P, et al. The economic burden of TB-affected households in DR Congo. Int J Tuberc Lung Dis. 2021;25(11):923–32. doi: 10.5588/ijtld.21.0182. PubMed PMID: 34686235; PubMed Central PMCID: PMCPMC8544924.

19. Rafai E, Underwood F, Delai M, Tuiwawa E, Daulako M, Nawadra-Taylor V, et al. National tuberculosis patient cost survey in Fiji 2017. 2020.

20. Ahmad RA, Probandari AN, Dewi C, Hafidz As Shidieq F, Satriani A, Widjanarko B, et al. Indonesia Tuberculosis Patient Cost Survey 2020. 2022.

21. Republic of Kenya Ministry of Health. The First Kenya Tuberculosis Patient Cost Survey. 2018.

22. Chittamany P, Yamanaka T, Suthepmany S, Sorsavanh T, Siphanthong P, Sebert J, et al. First national tuberculosis patient cost survey in Lao People's Democratic Republic: Assessment of the financial burden faced by TB-affected households and the comparisons by drug-resistance and HIV status. PLoS One. 2020;15(11):e0241862. Epub 20201112. doi: 10.1371/journal.pone.0241862. PubMed PMID: 33180777; PubMed Central PMCID: PMCPMC7660466.

23. Aung ST, Thu A, Aung HL, Thu M. Measuring Catastrophic Costs Due to Tuberculosis in Myanmar. Trop Med Infect Dis. 2021;6(3). Epub 20210714. doi: 10.3390/tropicalmed6030130. PubMed PMID: 34287379; PubMed Central PMCID: PMCPMC8293353.

24. Florentino JL, Arao RML, Garfin AMC, Gaviola DMG, Tan CR, Yadav RP, et al. Expansion of social protection is necessary towards zero catastrophic costs due to TB: The first national TB patient cost survey in the Philippines. PLoS One. 2022;17(2):e0264689. Epub 20220228. doi: 10.1371/journal.pone.0264689. PubMed PMID: 35226705; PubMed Central PMCID: PMCPMC8884492.

25. Muttamba W, Tumwebaze R, Mugenyi L, Batte C, Sekibira R, Nkolo A, et al. Households experiencing catastrophic costs due to tuberculosis in Uganda: magnitude and cost drivers. BMC Public Health. 2020;20(1):1409. Epub 20200916. doi: 10.1186/s12889-020-09524-5. PubMed PMID: 32938411; PubMed Central PMCID: PMCPMC7493412.

26. Nhung NV, Hoa NB, Anh NT, Anh LTN, Siroka A, Lonnroth K, et al. Measuring catastrophic costs due to tuberculosis in Viet Nam. Int J Tuberc Lung Dis. 2018;22(9):983–90. doi: 10.5588/ijtld.17.0859. PubMed PMID: 30092862.

27. Tiemersma EW, van der Werf MJ, Borgdorff MW, Williams BG, Nagelkerke NJD. Natural history of tuberculosis: duration and fatality of untreated pulmonary tuberculosis in HIV negative patients: a systematic review. PLOS ONE. 2011;6(4):e17601–e. doi: 10.1371/journal.pone.0017601.

28. Ragonnet R, Flegg JA, Brilleman SL, Tiemersma EW, Melsew YA, McBryde ES, et al. Revisiting the Natural History of Pulmonary Tuberculosis: A Bayesian Estimation of Natural Recovery and Mortality Rates. Clin Infect Dis. 2021;73(1):e88–e96. doi: 10.1093/cid/ciaa602. PubMed PMID: 32766718.

29. Frascella B, Richards AS, Sossen B, Emery JC, Odone A, Law I, et al. Subclinical Tuberculosis Disease—A Review and Analysis of Prevalence Surveys to Inform Definitions, Burden, Associations, and Screening Methodology. Clinical Infectious Diseases. 2020;73(3):e830–e41. doi: 10.1093/cid/ciaa1402.

30. Glaziou P, Arinaminpathy N, Dodd PJ, Dean A, Floyd K. Methods used by WHO to estimate the global burden of TB disease. Global Programme on Tuberculosis and Lung Health (GTB): World Health Organization, 2023.

31. World Health Organization. National surveys of costs faced by tuberculosis patients and their households 2015-2021: World Health Organization; 2022.

32. Griffin JT, Bhatt S, Sinka ME, Gething PW, Lynch M, Patouillard E, et al. Potential for reduction of burden and local elimination of malaria by reducing Plasmodium falciparum malaria transmission: a mathematical modelling study. Lancet Infect Dis. 2016;16(4):465–72. Epub 20160120. doi: 10.1016/S1473-3099(15)00423-5. PubMed PMID: 26809816; PubMed Central PMCID: PMCPMC5206792.

33. Camponovo F, Bever CA, Galactionova K, Smith T, Penny MA. Incidence and admission rates for severe malaria and their impact on mortality in Africa. Malar J. 2017;16(1):1. Epub 20170103. doi: 10.1186/s12936-016-1650-6. PubMed PMID: 28049519; PubMed Central PMCID: PMCPMC5209951.

34. Patouillard E, Griffin J, Bhatt S, Ghani A, Cibulskis R. Global investment targets for malaria control and elimination between 2016 and 2030. BMJ Glob Health. 2017;2(2):e000176. Epub 20170516. doi: 10.1136/bmjgh-2016-000176. PubMed PMID: 29242750; PubMed Central PMCID: PMCPMC5584487.
