# Supplementary material for: The benefits of investments to combat HIV, tuberculosis, and malaria for primary healthcare from 2000 to 2023: An economic modeling analysis
Source: PLoS Med. 2026 Apr 8;23(4):e1005036. doi: 10.1371/journal.pmed.1005036 (PMC13061260; doi:10.1371/journal.pmed.1005036)
Supplement: S1 CHEERS Checklist — (DOCX) [file pmed.1005036.s002.docx]

**The benefits of investments to combat HIV, tuberculosis and malaria for primary health care from 2000 to 2023: an economic modeling analysis**

# CHEERS 2022 Checklist

| **Topic** | **No.** | **Item** | **Location where item is reported** |
| --- | --- | --- | --- |
| **Title** |  |  |  |
|  | 1 | Identify the study as an economic evaluation and specify the interventions being compared. | Title, Page 1 |
| **Abstract** |  |  |  |
|  | 2 | Provide a structured summary that highlights context, key methods, results, and alternative analyses. | Abstract, Page 2 |
| **Introduction** |  |  |  |
| **Background and objectives** | 3 | Give the context for the study, the study question, and its practical relevance for decision making in policy or practice. | Introduction, Pages 5-6 |
| **Methods** |  |  |  |
| **Health economic analysis plan** | 4 | Indicate whether a health economic analysis plan was developed and where available. | Methods (Analytic summary) |
| **Study population** | 5 | Describe characteristics of the study population (such as age range, demographics, socioeconomic, or clinical characteristics). | Methods, Lines 107-112 |
| **Setting and location** | 6 | Provide relevant contextual information that may influence findings. | Methods, Lines 114-131 |
| **Comparators** | 7 | Describe the interventions or strategies being compared and why chosen. | Methods, Lines 114-131 |
| **Perspective** | 8 | State the perspective(s) adopted by the study and why chosen. | Introduction, Lines 88-96 |
| **Time horizon** | 9 | State the time horizon for the study and why appropriate. | Introduction, Line 101; Methods, Line 114 |
| **Discount rate** | 10 | Report the discount rate(s) and reason chosen. | NA |
| **Selection of outcomes** | 11 | Describe what outcomes were used as the measure(s) of benefit(s) and harm(s). | Methods, Lines 120-122 |
| **Measurement of outcomes** | 12 | Describe how outcomes used to capture benefit(s) and harm(s) were measured. | Methods, Lines 122-131 |
| **Valuation of outcomes** | 13 | Describe the population and methods used to measure and value outcomes. | Methods, Line 107, Lines 136-252 |
| **Measurement and valuation of resources and costs** | 14 | Describe how costs were valued. | Methods, Lines 136-252 |
| **Currency, price date, and conversion** | 15 | Report the dates of the estimated resource quantities and unit costs, plus the currency and year of conversion. | Methods, Line 252 |
| **Rationale and description of model** | 16 | If modelling is used, describe in detail and why used. Report if the model is publicly available and where it can be accessed. | Methods, Lines 151-252 |
| **Analytics and assumptions** | 17 | Describe any methods for analysing or statistically transforming data, any extrapolation methods, and approaches for validating any model used. | Appendix, Pages 1-2 |
| **Characterising heterogeneity** | 18 | Describe any methods used for estimating how the results of the study vary for subgroups. | NA |
| **Characterising distributional effects** | 19 | Describe how impacts are distributed across different individuals or adjustments made to reflect priority populations. | NA |
| **Characterising uncertainty** | 20 | Describe methods to characterise any sources of uncertainty in the analysis. | Methods (Sensitivity analyses) |
| **Approach to engagement with patients and others affected by the study** | 21 | Describe any approaches to engage patients or service recipients, the general public, communities, or stakeholders (such as clinicians or payers) in the design of the study. | NA |
| **Results** |  |  |  |
| **Study parameters** | 22 | Report all analytic inputs (such as values, ranges, references) including uncertainty or distributional assumptions. | Appendix, Pages 7-9 |
| **Summary of main results** | 23 | Report the mean values for the main categories of costs and outcomes of interest and summarise them in the most appropriate overall measure. | Results, Lines 309-371 |
| **Effect of uncertainty** | 24 | Describe how uncertainty about analytic judgments, inputs, or projections affect findings. Report the effect of choice of discount rate and time horizon, if applicable. | Results (Sensitivity analyses) |
| **Effect of engagement with patients and others affected by the study** | 25 | Report on any difference patient/service recipient, general public, community, or stakeholder involvement made to the approach or findings of the study | NA |
| **Discussion** |  |  |  |
| **Study findings, limitations, generalisability, and current knowledge** | 26 | Report key findings, limitations, ethical or equity considerations not captured, and how these could affect patients, policy, or practice. | Discussion, Pages 19-23 |
| **Other relevant information** |  |  |  |
| **Source of funding** | 27 | Describe how the study was funded and any role of the funder in the identification, design, conduct, and reporting of the analysis | End of manuscript (Funding Information and Financial Disclosure) |
| **Conflicts of interest** | 28 | Report authors conflicts of interest according to journal or International Committee of Medical Journal Editors requirements. | End of manuscript (Conflict of Interests) |

Husereau D, Drummond M, Augustovski F, de Bekker-Grob E, Briggs AH, Carswell C, Caulley L, Chaiyakunapruk N, Greenberg D,Loder E, Mauskopf J, Mullins CD, Petrou S, Pwu RF, Staniszewska S; CHEERS 2022 ISPOR Good Research Practices Task Force.Consolidated Health Economic Evaluation Reporting Standards 2022 (CHEERS 2022) Statement: Updated Reporting Guidance for HealthEconomic Evaluations. BMJ. 2022;376:e067975.

The checklist is Open Access distributed in accordance with the terms of the Creative Commons Attribution (CC BY 4.0) license, whichpermits others to distribute, remix, adapt and build upon this work, for commercial use, provided the original work is properly cited. See:http://creativecommons.org/licenses/by/4.0/.

For more information, please see: <https://doi.org/10.1136/bmj-2021-067975>
